# The Cell Wall Integrity Receptor Mtl1 Contributes to Articulate Autophagic Responses When Glucose Availability Is Compromised

**DOI:** 10.3390/jof7110903

**Published:** 2021-10-26

**Authors:** Sandra Montella-Manuel, Nuria Pujol-Carrion, Maria Angeles de la Torre-Ruiz

**Affiliations:** Cell Signalling in Yeast Unit, Department of Basic Medical Sciences, Institut de Recerca Biomèdica de Lleida (IRBLleida), University of Lleida, 25198 Lleida, Spain; sandra.montella@udl.cat (S.M.-M.); nuria.pujol@udl.cat (N.P.-C.)

**Keywords:** cell wall integrity (CWI), Mtl1, autophagy, glucose, mitophagy, *Saccharomyces cerevisiae*

## Abstract

Mtl1protein is a cell wall receptor belonging to the CWI pathway. Mtl1 function is related to glucose and oxidative stress signaling. In this report, we show data demonstrating that Mtl1 plays a critical role in the detection of a descent in glucose concentration, in order to activate bulk autophagy machinery as a response to nutrient deprivation and to maintain cell survival in starvation conditions. Autophagy is a tightly regulated mechanism involving several signaling pathways. The data here show that in *Saccharomyces* *cerevisiae*, Mtl1 signals glucose availability to either Ras2 or Sch9 proteins converging in Atg1 phosphorylation and autophagy induction. TORC1 complex function is not involved in autophagy induction during the diauxic shift when glucose is limited. In this context, the *GCN2* gene is required to regulate autophagy activation upon amino acid starvation independent of the TORC1 complex. Mtl1 function is also involved in signaling the autophagic degradation of mitochondria during the stationary phase through both Ras2 and Sch9, in a manner dependent on either Atg33 and Atg11 proteins and independent of the Atg32 protein, the mitophagy receptor. All of the above suggest a pivotal signaling role for Mtl1 in maintaining correct cell homeostasis function in periods of glucose scarcity in budding yeast.

## 1. Introduction

Carbon sources have a major impact on *Saccharomyces cerevisiae* metabolism and also affect longevity. Yeast uses a fermentative metabolism when the preferred carbon source, glucose, is abundant and ethanol, organic acids, and ATP are accumulated. Glucose limitation induces slowing of growth, contributing to the switch to respiratory metabolism, the hallmark of the diauxic shift, which along with other metabolic configurations prepares cells for the stationary phase and the process of chronological ageing [1]. Budding yeast chronological life span (CLS) forced by glucose restriction is also dependent on the availability of other nutrients in the culture media, such as amino acids or nitrogen among others [2,3,4,5]. In a simple way, glucose is a promotor of ageing through the activation of the Ras-cAMP-PKA pathway and TORC1-Sch9 [6,7,8] pathways, whereas amino acids operate through Gcn2 protein [9,10,11,12] as well as through activation of the TORC1 complex, upstream regulators of the Sch9 protein [13].

The TORC1 and Ras/PKA pathways are both negative and independent regulators of autophagy [13,14,15,16]. Sch9 downregulates autophagy independently and coordinately with the Ras/PKA pathway [17].

Ras proteins are essential for growth in fermentable carbon sources such as glucose. In that context, Ras proteins trigger the synthesis of cAMP and activation of the PKA pathway upon binding to the inhibitor Bcy1 protein. The absence of Ras proteins does not allow growth in non-fermentative carbon sources (see reviews [18,19]).

The sucrose non-fermenting protein kinase, Snf1, is the yeast orthologue of mammalian AMPK, and is important for cell adaptation to glucose limitation. Snf1 is a key component of the main glucose repression pathway in yeast and controls genes involved in alternative carbon sources and metabolism. However, regulation of adaptation to glucose limitation is the main role of the SNF1 complex [20,21]. Snf1 also plays a role in autophagy [22]; its negative regulation is required to downregulate autophagy under certain conditions of nutritional limitation [23].

Mtl1 protein is a transmembrane protein cell wall mechano-sensor [24,25], part of the cell wall integrity pathway (CWI) with structural similarity to its paralogue Mid2 protein [26,27]. Mtl1 is also required to activate a stress response towards TORC1 and Ras/PKA signaling pathways under conditions of both oxidative stress and glucose starvation [28]. In addition, this cell wall receptor plays a role in Cyclin C localization and programmed cell death [29], as well as in the preservation of mitochondrial integrity and life span by regulating TORC1, Sch9, Slt2, and PKA [30].

The CWI is involved in sensing and transducing a wide variety of stimuli (see reviews [31,32]), including both nutritional and oxidative stresses [27,30,33]. In this pathway, a wide number of sensors are specialized in the detection of different stresses [25,34], which converge on the GTPase Rho1 to subsequently activate Pkc1 protein (see reviews [31,32]). Pkc1 in turn phosphorylates the Bck1 protein, the MAPKKK, thus activating the MAPKK module composed of Mkk1/Mkk2 proteins leading to dual phosphorylation and activation of the last member of the pathway, the MAPK Slt2 protein, whose double phosphorylation is impaired in the mutant *mtl1* [30].

Nutrient limitation strongly induces macroautophagy [23,35,36,37] to accomplish two main objectives; one is to detoxify and the second is to recycle components to build newly synthetized molecules.

Macroautophagy is a process in which several components (damaged or superfluous organelles, cytoplasmic elements, microorganisms…) are engulfed within cytosolic double membrane vesicles named autophagosomes. The outer membrane of the phagosome fuses to the vacuolar membrane releasing a spherical body termed autophagic body, that is digested by hydrolases releasing the breakdown products back to the cytosol to be recycled by cells [38]. Morphological and structural characteristics of autophagy are highly conserved from yeast to humans.

The signaling network governing life span usually converges in the autophagic machinery [17,23,39]. In general, nutrient deprivation impinges on Atg13 dephosphorylation that triggers Atg1 kinase activity then leading to the formation of the complex Atg13/Atg1/Atg17/Atg29/Atg31 activating the autophagy process [40,41]. Atg7 is an E1-like enzyme essential for macroautophagy since it is part of the Atg8-Ibl conjugation system [42,43]. Mutants defective in autophagy display shorter life spans [44,45].

Autophagy entails nonselective engulfment of cytoplasmic components, but there are also other types of autophagy that selectively degrade specific cellular elements (see review [46]), such is the case of mitophagy. Mitophagy clears dysfunctional mitochondria and impinges on cellular function by promoting respiration proficiency during the process of ageing (see review [47]). Selective mitophagy requires the function of the Atg32 protein as a mitochondrial receptor and its binding to the adaptor Atg11, which interacts with the Atg8 protein in the phagophore inner surface (see review [48]). Atg33 is a yeast protein located at the outer mitochondrial membrane, its absence suppressed mitophagy in post-log cultures, however, its precise role in mitophagy is still controversial [49].

In this report, we show that bulk autophagy is highly induced during the transition to diauxic shift in a manner totally dependent on glucose and amino acids availability. The Mtl1 cell wall receptor is essential to sense glucose concentration and transmit the signal to both Ras2 and Sch9 to phosphorylate Atg1 and to activate the macroautophagic machinery. Gcn2 is the amino acid sensor. Both Mtl1 and Gcn2 operate independently of TORC1 in the signaling process leading to the activation of bulk autophagy. Moreover, Mtl1 is also relevant for mitochondrial clearance dependent on Atg33 and the inactivation of either Ras2 or Sch9 in response to glucose exhaustion.

## 2. Materials and Methods

### 2.1. Yeast Strains and Plasmids

*Saccharomyces cerevisiae* strains are listed in Table 1. All the strains named GSL are derivatives of the CML128 background. New null mutants described in this study were obtained by a one-step disruption method that used the *Nat*Mx4 or *Kan*Mx4 cassettes [50]. Strains GSL197, 198, 199, 200, 201, 202, 226, 265, 279, 297, 352, 370, 382, 414, and 415 were constructed upon integration of plasmid pGFP–Atg8 (original name: pHab142), previously digested with Stu1, in the *URA3* gene. Strains GSL372 and 416 were constructed upon integration of plasmid pAtg1-HA previously digested with BstEII. The plasmid pAtg1-HA was obtained upon Atg1 cloning into the Pme1 and PstI sites of the integrative vector pMM351 [51].

Plasmid descriptions are listed in Table 2. Each particular ORF was amplified by PCR from genomic DNA to be directionally cloned in the specific plasmid.

### 2.2. Media, Growth Conditions and Reagents

Yeasts were grown at 30 °C in SD medium (2% glucose, 0.67% yeast nitrogen base that lacked the corresponding amino acids for plasmid maintenance) plus amino acids [59].

Glucose depletion consisted of SD medium without glucose plus amino acids. Nitrogen depletion consisted of SD medium whose nitrogen base component was free of amino acids and ammonium sulphate plus amino acids. Amino acid depletion consisted of SD medium without adding amino acids. Glycerol medium (3% glycerol, 0.67% yeast nitrogen base that lacked the corresponding amino acids) plus amino acids. Sucrose medium (2% sucrose, 0.67% yeast nitrogen base that lacked the corresponding amino acids) plus amino acids. Fructose medium (2% fructose, 0.67% yeast nitrogen base that lacked the corresponding amino acids) plus amino acids.

Glucose was added as α-D-glucose monohydrate (Serva, Heidelberg, Germany 22720.01) at a final concentration of 2%; Amino acids were added at concentrations: 60 mg/mL Leucine, 20 mg/mL Histidine and 20 mg/mL Tryptophan. Nitrogen was added as Yeast Nitrogen Base w/o Amino Acids (Difco, Franklin Lakes, NJ, USA, 291940) at a final concentration of 0.67%. Sucrose was added as Sucrose (Sigma, Saint Louis, MI, USA, S0389) at a final concentration of 2% and Fructose was added as D-Fructose (Sigma, Saint Louis, MI, USA, 47740) at a final concentration of 2%.

We present a list of reagents detailing final concentrations in culture media and from which company they were purchased: N-Acetyl cysteine (NAC) 5 mM (Sigma, Saint Louis, MI, USA, A9165); FM4-64 30 μg/μL (Invitrogen, Waltham, MA, USA, T-3166); Rapamycin 200 ng/mL (Sigma, Saint Louis, MI, USA, R0395); ATP 200 mM (Sigma, Saint Louis, MI, USA, A1852); and Dihydroethidium (DHE) 50 μM (Sigma, Saint Louis, MI, USA, D7008). Cell cultures were exponentially grown at 600 nm [O.D_600_] of 0.6. Iron was added as ammonium iron (III) sulphate hexahydrate [NH_4_Fe(SO_4_)_2_·6H_2_O] (+Fe; Sigma, Saint Louis, MI, USA, F1543) at a final concentration of 10 mM.

### 2.3. Vacuole and Dihydroethidium Staining

For vacuole visualization, cells were stained with the fluorescent styryl dye FM4-64 (N-(3-triethylammoniumpropyl)-4-(p-diethylaminophenylhexatrienyl) pyridinium dibromide. To determine cellular oxidation, we used dihydroethidium (DHE). Both protocols were previously described by our group in [60].

### 2.4. Cell Survival and Chronological Life Span

To assay cell viability cells were grown to mid-log phase OD_600_:0.6 in SD medium supplemented with the required amino acids. Viability was registered through serial dilutions and plated by triplicate onto YPD plates.

We measured the chronological life span (CLS) in the different strains based on the survival of populations of non-dividing yeast cells according to [61]. The viability was scored by counting the number of cells able to form colonies, CFU (colony-forming units). Cultures were started at an OD_600_:0.6. The same number of cells collected from each culture were plated in triplicated into YPD plates and allowed to grow at 30 °C for 3–4 days. CLS curves were plotted with the corresponding averages and standard deviations from three independent experiments.

### 2.5. Protein Extraction and Immunoblot Analyses

We followed an identical procedure as described in [23]. Total yeast protein extracts were prepared as previously described in [61]. The antibodies for Western blotting were as follows: anti-HA 3F10 (No. 12158167001; Roche Applied Science, Penzberg, Germany), used at a dilution of 1:2000 in 0.25% non-fat milk, and the corresponding secondary was goat anti-Rat IgG horseradish peroxidase conjugate (No. AP136P, Millipore, Burlington, MA, USA). Anti-GFP (No. 632381; Living Colors Mountain View, CA, USA) was used at a dilution of 1:2000 and anti-Phospho-glycerate kinase 1 (anti-PGK1) (459250, Invitrogen, Waltham, MA, USA) was used at a dilution 1:1200, both with the secondary antibody anti-Mouse horseradish peroxidase conjugate (LNA931v/AG, GE Healthcare, Chicago, IL, USA) and anti-Phospho-AMPKα (Thr172) (167253S, Cell Signalling, Danvers, MA, USA) at a dilution of 1:1000 with the secondary antibody anti-rabbit horseradish peroxidase conjugate (LNA934v/AG, GE Healthcare, Chicago, IL, USA). They were used as indicated by the manufacturers.

The protein–antibody complexes were visualized by enhanced chemiluminescence, using the Supersignal substrate (Pierce, Waltham, MA, USA) in a Chemidoc (Roche Applied Science, Penzberg, Germany).

For all the figures: We used anti-PGK1 to detect PGK1, selected as a loading control in all the Western blots shown in this study. For Western blots in this paper, we have selected representative samples.

### 2.6. Autophagy Detection

Autophagy progression is monitored through several complementary approaches, such as the immunological detection of GFP accumulation from GFP–Atg8 genomic fusion which is delivered to the vacuole to be degraded once autophagy is induced. The GFP moiety is very resistant to proteolysis compared to Atg8 which is rapidly degraded in the vacuole. Therefore, detection of free GFP processed from GFP–Atg8 is a very reliable tool to measure levels of complete autophagy through the autophagic flux [62], that is delivery and turnover of the cargo in the vacuole. Autophagic flux is the ratio of free GFP/GFP–Atg8+free GFP quantified upon Western blot detection by using anti-GFP antibody [23]. Another complementary approach is the microscopic observation of GFP accumulation in vacuoles. For all microscope panels, we have used a representative image of either log or one day samples in order to identify GFP–Atg8 localization. In general, both assays are sufficient as evidence of autophagy activity.

In some particular occasions we also use an alternative approach, consisting of measuring *pho8*Δ60 enzymatic activity to determine nonspecific autophagy, as described by Noda and Klionsky [63] and modified by Guedes et al. [54].

### 2.7. Glucose Determination

We followed the directions detailed in [64].

### 2.8. Statistical Analysis

We followed the same procedure as described in Montella et al. [23]. Error bars in the histograms represent the standard deviation (SD) calculated from three independent experiments. Significance of the data was determinate by *p*-values from a Student’s unpaired *t*-test denoted as follows: * = 0.05 > *p* > 0.01; ** = 0.01 > *p* > 0.001; *** = 0.001 > *p* > 0.0001; **** = *p* > 0.0001.

## 3. Results

### 3.1. Glucose, Amino Acids, Nitrogen and Iron Deprivation Determine the Induction of Bulk Autophagy during Diauxic Transition

**Figure 1:**
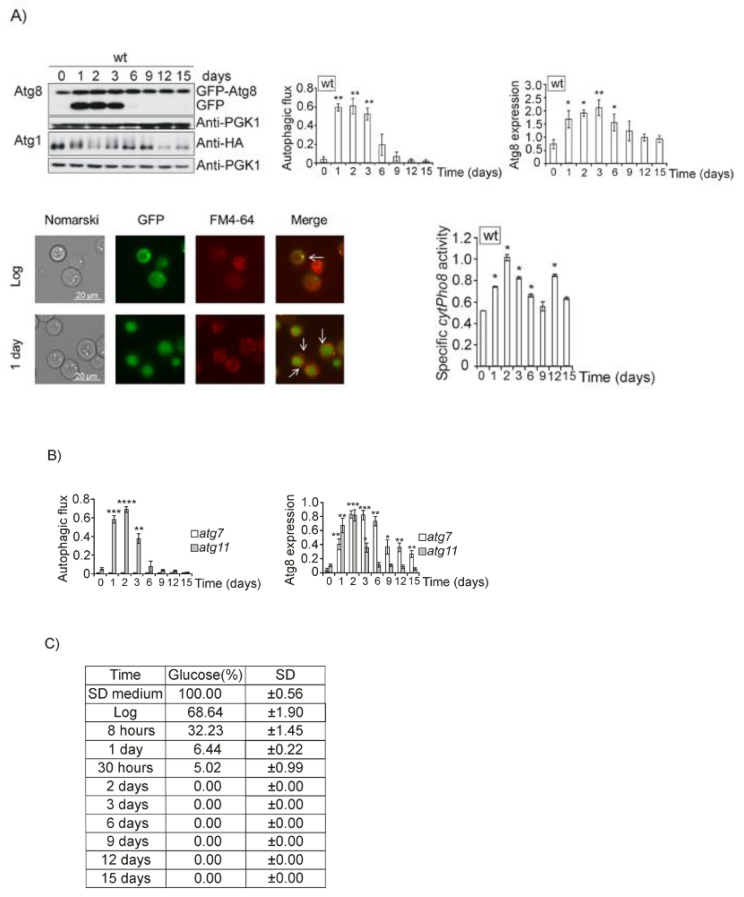

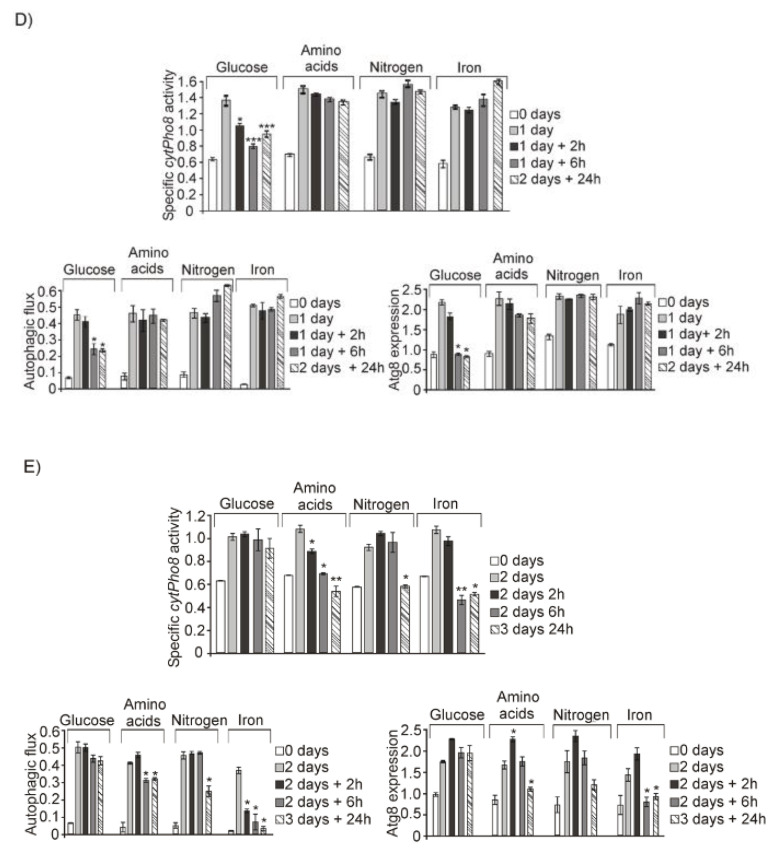
Sequential decreases of glucose and amino acids activates bulk autophagy during the diauxic shift in *Saccharomyces cerevisiae*. (**A**) wt cultures in which the fusion protein GFP–Atg8 or Atg1HA were integrated, were grown to log phase (OD_600_: 0.6) in SD medium at 30 °C. Aliquots were collected at the indicated times for total protein extraction and Western blot analysis. GFP–Atg8 was monitored using an anti-GFP antibody. We used anti-PGK1 to detect Pgk1 as a loading control. Microscopic observation of GFP–Atg8 was carried out using a fluorescence microscope. GFP vacuolar accumulation was also determined upon the use of the fluorescent dye FM4-64. Autophagic flux was calculated as the ratio of free GFP and total GFP–Atg8 in the samples. Total GFP–Atg8 was determined as the addition of the form GFP–Atg8 and the band corresponding to free GFP, as a result of Atg8 vacuolar degradation, both detected by Western blot. Enzymatic autophagy activity was measured by using the alkaline phosphatase assay in the strain BY4741*pho8*Δ expressing a plasmid with the inactive Pho8 proenzyme targeted to the cytosol. Values of Atg1 proteins were determined upon Western blot analysis using anti-HA antibody. (**B**) *atg7* and *atg11* strains expressing the fusion protein GFP–Atg8 were grown in the same conditions as described in A. Autophagic flux and total Atg8 expression were determined as in (**A**). (**C**) Glucose content in the culture medium (%) was determined in wt cultures growing in SD media at 30 °C at the days indicated in the table for a total period of 15 days. Glucose in the sterile media, SD, at a final concentration of 2%, is the equivalent of 100% in the table. (**D**) wt cells bearing GFP–Atg8 in their genome were exponentially grown at OD_600_:0.6 at 30 °C in SD media and a sample was collected for analysis. Upon one day of culture, 2% glucose, amino acids (60 mg/mL leucine, 20 mg/mL histidine, and 20 mg/mL tryptophan), 0.67% nitrogen or 10 mM iron, were respectively added to the cultures and samples were collected at 2, 6, and 24 h to perform *pho8*Δ60 enzymatic assays. Autophagic flux and GFP–Atg8 total expression were determined as previously detailed in A. Enzymatic autophagic activity was determined as in (**A**). (**E**) As in (**D**), but results correspond to two days of growth. Error bars in the histograms represent the standard deviation (SD) calculated from three independent experiments. Significance of the data was determinate by *p*-values from a Student’s unpaired *t*-test denoted as follows: * = 0.05 > *p* > 0.01; ** = 0.01 > *p* > 0.001; *** = 0.001 > *p* > 0.0001; **** = *p* > 0.0001.

In a previous paper we demonstrated that autophagy is required for normal life span extension [23]. We wanted to determine which pathways are involved in autophagy regulation in the process of ageing. To start our analysis, we took daily samples from log phase (day 0) till day 15, following a standard CLS analysis. We used SD medium to avoid the addition of excess amino acids and thus did not affect the metabolism of the yeast cells. In Figure 1A we can observe that there is a great induction of autophagy and autophagic flux when cells reach the diauxic shift upon one day of growth, which is maintained and gradually decreases until day 6. The induction of autophagic flux is related with the phosphorylation of Atg1 protein, hence, Atg1 receives the starvation signal in order to induce autophagy during the diauxic and posdiauxic shifts, and also with the enzymatic activity determined by the *pho8*Δ60 assay. Microscopic observation of the cultures confirmed the former results, since free GFP derived from GFP–Atg8 fusion protein was accumulated inside vacuoles which appear colored in green and surrounded by a red membrane stained by the fluorescent styryl dye FM4-64. In order to ascertain whether our results were compatible with bulk or selective autophagy we repeated this experiment in the mutants *atg7* (involved in general autophagy) and *atg11* (representing of selective autophagy) (Figure 1B and Figure S1B). Our results demonstrate that free GFP liberated from GFP–Atg8 fusion protein and detected both in Western blot and in the fluorescence microscopy indicated bulk autophagy and was independent of any type of selective autophagy.

One day of culture in SD media is the transition between a fermentative to respiratory metabolism, the diauxic shift, a metabolic regulatory checkpoint determinant in the process of ageing. At this point, we could observe that glucose is nearly exhausted in the culture media (Figure 1C). Consequently, we performed refeeding experiments upon one day of culture and observed that only upon 6 h of glucose addition autophagy (determined by *pho8*Δ60 specific activity, identification of free GFP by Western blot, and in vivo fluorescence identification of vacuolar accumulation of GFP) significantly decreased, concluding that a severe decrease in glucose concentration provoked the induction of autophagy, and that there is an increase in autophagy (Figure 1D and Figure S1C). We carried out the same strategy with other nutrients which could also be limiting: amino acids, nitrogen and iron. Upon refeeding of iron, nitrogen, and amino acids, we observed that one-day refeeding did not provoke changes in autophagy (Figure 1D and Figure S1C), however upon two days of culture we observed a clear descent in GFP accumulation caused by amino acid replenishment (Figure 1E,D). Both nitrogen and iron replenishment provoked a descent in autophagy upon two days of culture.

### 3.2. Both Mtl1 and Gcn2 Control Autophagy Induction during Diauxic Transition

In this context, we decided to explore the signaling pathways that could be involved in signaling autophagy when cells age.

**Figure 2:**
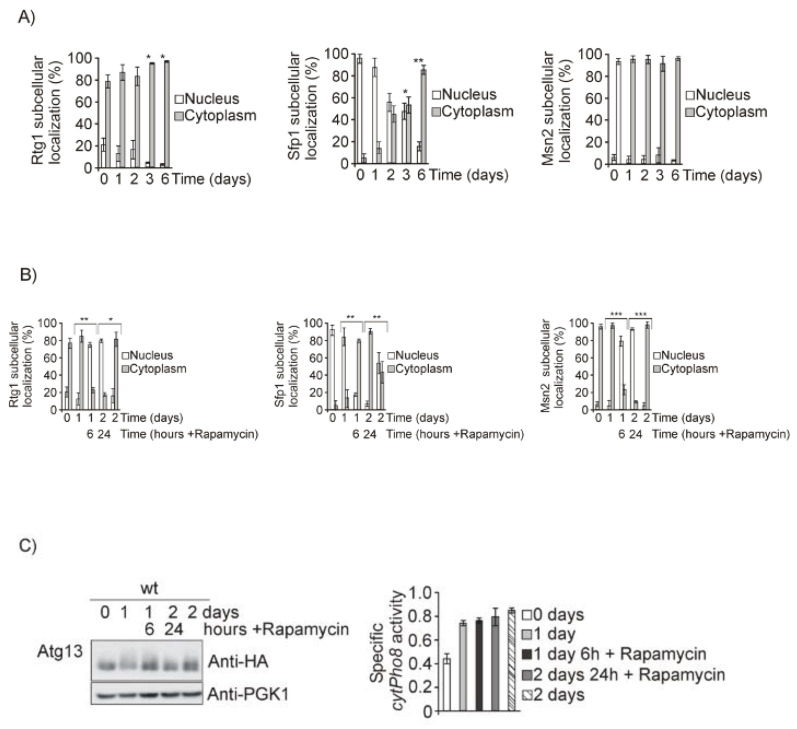
TORC1 is not inactivated during diauxic and posdiauxic shifts. (**A**) wt strains transformed with the plasmids Rtg1GFP, Sfp1GFP, or Msn2GFP respectively, were grown at 30 °C in SD media for the times indicated in the figures. Aliquots were collected for in vivo observation in the fluorescence microscope. Histograms represent the percentages of in vivo nuclear or cytoplasmic localization out of more than 1000 cells. (**B**) Cultures in A were treated with rapamycin (200 ng/mL) on day one of culture for 6 h and aliquots were collected for in vivo observation in the fluorescence microscope. Histograms are performed as in (**A**). (**C**) A wt strain expressing Atg13HA was exponentially grown at 30 °C in SD media. Rapamycin was added to the culture upon one day of growth at 200 ng/mL and samples were collected upon 6 and 24 h of exposure to the drug for total protein extraction, Western blot analysis, and identification of Atg13HA using anti-HA antibody. Autophagic enzymatic activity was determined through the alkaline phosphatase assay, as in (Figure 1A). Error bars in the histograms represent the standard deviation (SD) calculated from three independent experiments. Significance of the data was determinate by *p*-values from a Student’s unpaired *t*-test denoted as follows: * = 0.05 > *p* > 0.01; ** = 0.01 > *p* > 0.001; *** = 0.001 > *p* > 0.0001.

We considered the possibility that amino acid and nitrogen depletion would provoke TORC1 inactivation at least partly. We analyzed TOR function by means of the readouts Rtg1, Sfp1, Msn2. Under TORC1 inactivation, Rtg1 and Msn2 were localized to the nucleus, whereas Sfp1 was located in the cytoplasm, confirming that Tor1 is not inactivated in our model (Figure 2A). In addition, we added rapamycin, a drug that specifically inactivates TORC1, to the previous cultures as a control, and we observed that Rtg1 and Msn2 localized to the nucleus and Sfp1 was located in the cytoplasm (Figure 2B). Moreover, rapamycin treatment caused a decrease in Atg13 phosphorylation and autophagy values calculated upon Pho8 assay did not increase, supporting the conclusion that TORC1 is not completely inactivated upon diauxic shift in the conditions of our study (Figure 2C).

**Figure 3:**
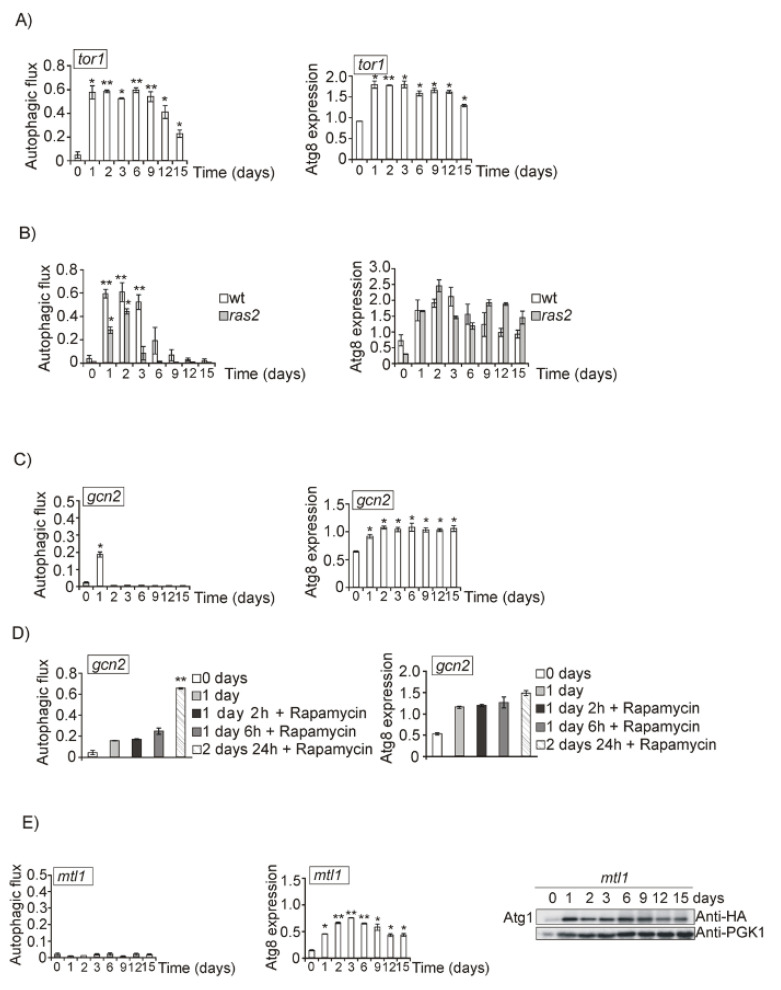

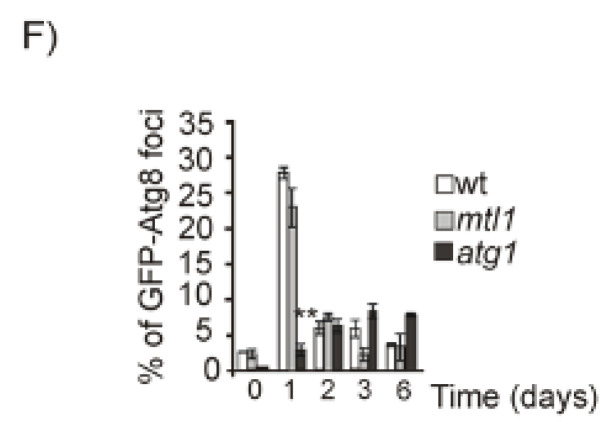
Mtl1 and Gcn2 control autophagy induction during glucose and amino acid starvation. Growth conditions, total Atg8 determination and autophagic flux determined in: (**A**) *tor1* mutant expressing GFP–Atg8; (**B**) wt strain and *ras2* mutant expressing GFP–Atg8; and (**C**) *gcn2* mutant expressing GFP–Atg8, performed as described in Figure 1B. (**D**) *gcn2* bearing GFP–Atg8 was exponentially grown at 30 °C in SD plus amino acids. Rapamycin (200 ng/mL) was added to the cultures upon 1 day of growth and samples were subsequently collected upon 2, 6 and 24 h of exposure to the drug. Aliquots were treated as in Figure 1B. (**E**) Growth conditions, total Atg8 and autophagic flux determinations in *mtl1* culture expressing the fusion protein GFP–Atg8 was performed as in Figure 1B. Identification of Atg1 protein in *mtl1* cultures transformed with the plasmid Atg1HA was performed upon Western blot analysis using the anti-HA antibody as in Figure 1A. (**F**) Percentage of Atg8 foci quantified in the experiments, described in Figure 1A and Figure 3E, was calculated upon microscopic observation of more than 1000 cells. The axis label “% of GFP–Atg8 foci” refers to the percentage of cells with GFP–Atg8 foci. Error bars in the histograms represent the standard deviation (SD) calculated from three independent experiments. Significance of the data was determinate by *p*-values from a Student’s unpaired *t*-test denoted as follows: * = 0.05 > *p* > 0.01; ** = 0.01 > *p* > 0.001.

However, when *TOR1* was deleted we observed that autophagy (free GFP detected in the Western blot and fluorescence microscope accumulated in vacuoles) was extended to longer times (Figure 3A and Figure S2A), concomitant with longer life extension as described in [54]. Taken together, we conclude that TORC1 is not inactivated in our system during the transition between fermentative and respiratory metabolism, therefore it is not the main pathway responsible for the bulk autophagy induction.

We also explored a *ras2* mutant, since Ras2 is active in exponentially growing cells and becomes inactive as long as cells enter in respiratory metabolism and glucose becomes exhausted. *RAS2* deletion partially affected autophagy progression as compared to wt cultures (Figure 3B and Figure S2B). Since other nutrients become depleted upon the diauxic shift, we took into consideration the Gcn2/eIF2alpha pathway which becomes activated under amino acid and other nutrient starvation [65]. The Gcn2 pathway is activated upon diauxic shifting, indicating the moment in which amino acid concentrations significantly decreased in the culture media. According to our results, and coincident with the above mentioned replenishment results, day 2 of growth should be the moment in which cells become starved for amino acids. We observed that upon the second day, autophagy disappears when Gcn2 is deleted, however the absence of Gcn2 the burst in autophagy observed upon 1 day of growth was not affected (Figure 3C and Figure S2C). This result suggests that Gcn2 is required to induce macroautophagy upon two days of growth in SD medium, after the diauxic shift, probably due to a descent in the amino acid concentration one day after glucose starvation, suggesting that Gcn2 is not involved in autophagy signaling in response to carbon sources. Following this observation, we wanted to ascertain whether the regulatory function mediated by Gcn2 was dependent on TORC1. We treated *gcn2* mutant cultures on day 1 with rapamycin and observed induction of autophagy, suggesting that in the transition from fermentative to respiratory metabolism, TORC1 and *GCN2* are independent (Figure 3D and Figure S2D). As expected, this conclusion is consistent with the previous observation that TORC1 function is not inactivated during the CLS experiment; as a consequence of that, this pathway is not relevant for autophagy induction during ageing in our experimental conditions.

Our results suggest that glucose is the principal nutrient in the culture medium we use, whose decrease causes autophagy induction during the diauxic shift (1 day of growth) therefore, we decided to analyze in more depth the role that Mtl1 could be playing in this process. Mtl1 is a cell wall receptor belonging to the CWI pathway, involved in glucose signaling during the diauxic shift and stationary phase [28,30]. Interestingly, in the absence of Mtl1, autophagy is undetectable by Western blot, with Atg1HA phosphorylation or in vivo GFP–Atg8 microscopic accumulation through all experiments, from day 1 to 15 (Figure 3E and Figure S2E). In yeast, autophagy is initiated when the pre-autophagosome (PAS) structure is formed [66]. PAS can be detected in the fluorescence microscope as dotted accumulations of Atg proteins next to the vacuole. In *mtl1* diauxic cultures, PAS can be detected, as also observed in wt strain, and they are significantly higher than in the *atg1* mutant (Figure 3F), suggesting that Mtl1 does not block the initiation of the autophagy complex. This result suggests that Mtl1 is essential for receiving the signal of glucose concentration during the diauxic shift and to transmission of this signal to the autophagy machinery. We have also detected similar results when using alternative and fermentative carbon sources such as sucrose or fructose (Figure S2F).

### 3.3. Mtl1 CWI Cell Wall Receptor Signals Glucose Concentration to the Autophagy Machinery in a Manner Partly Dependent on Intracellular ATP Levels

**Figure 4:**
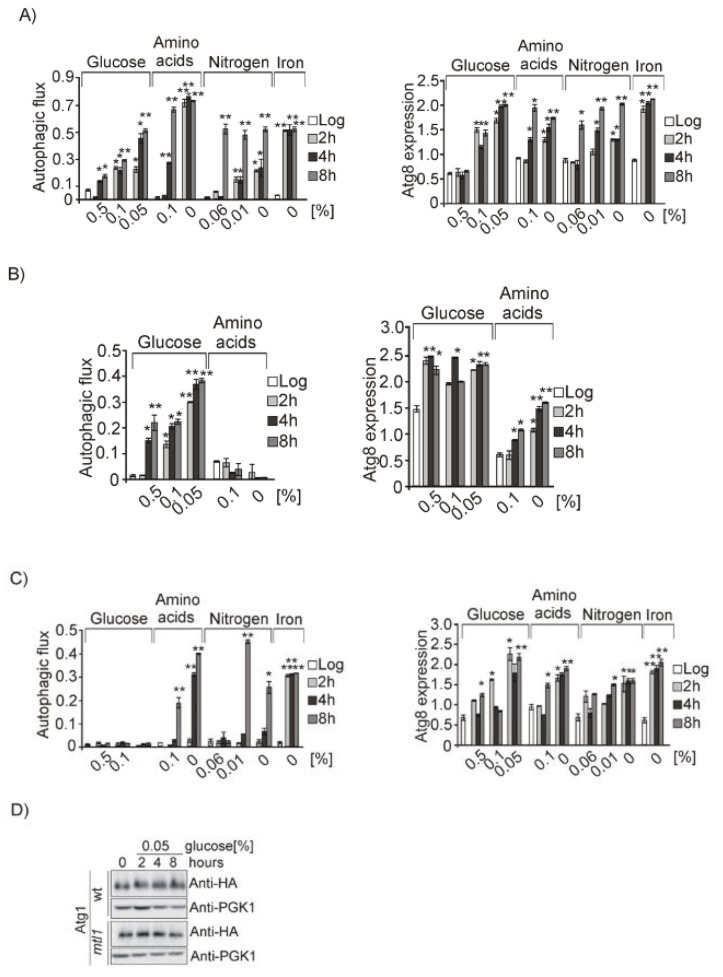
Mtl1 signals glucose limitation to autophagy machinery. (**A**) wt cells expressing GFP–Atg8 were exponentially grown in SD media. Aliquots were taken, washed, and transferred to different minimum media containing: 0.5, 0.1, or 0.05% glucose; 0.1 or 0% amino acids; 0.06, 0.01, or 0% nitrogen or medium without iron (0%). Autophagic flux and Atg8 expression were determined as in Figure 1B. The same experiments as in A were carried out in (**B**) *gcn2* mutant cultures expressing GFP–Atg8 and in a (**C**) *mtl1* strain expressing GFP–Atg8. (**D**) Atg1HA protein was identified by Western blot using anti-HA antibody, as in Figure 1A. Error bars in the histograms represent the standard deviation (SD) calculated from three independent experiments. Significance of the data was determinate by *p*-values from a Student’s unpaired *t*-test denoted as follows: * = 0.05 > *p* > 0.01; ** = 0.01 > *p* > 0.001; *** = 0.001 > *p* > 0.0001; **** = *p* > 0.0001.

In previous reports it has been demonstrated that several nutritional stressors (nitrogen, amino acids, iron…) cause the induction of autophagy. In order to analyze the specificity that Mtl1 could play in macroautophagy regulation, we used different nutrient concentrations: (glucose: 0.5%, 0.1%, 0.05% and 0%; amino acids: 0.1% and 0%; nitrogen: 0.06%, 0.01% and 0%; and iron: 0%). The decrease of each of the nutrients, glucose, amino acids, iron, or nitrogen concentrations induced the activation of macroautophagy in wt cells (Figure 4A and Figure S3A) along with corresponding Atg1 phosphorylation (Figure 4D). In addition, the absence of Gcn2 precluded autophagy in a manner only dependent on amino acid availability (Figure 4B and Figure S3B), whereas the absence of Mtl1 specifically abolished the glucose deprivation dependent autophagy (Figure 4C and Figure S3C), supported by a lack of Atg1 phosphorylation (Figure 4D). We demonstrated that glucose concentrations below 0.5% caused a clear induction of autophagy specifically mediated by Mtl1, as in *mtl1* mutants autophagy was not induced.

**Figure 5:**
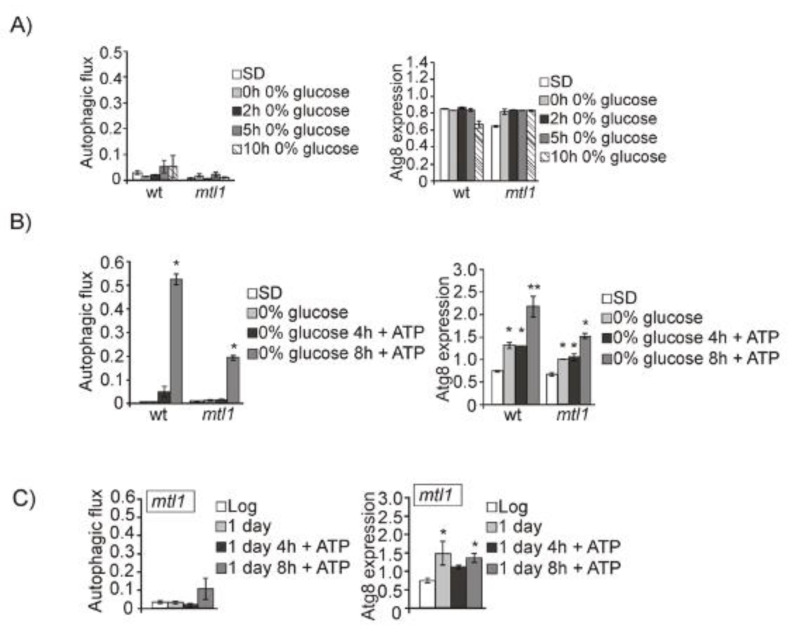

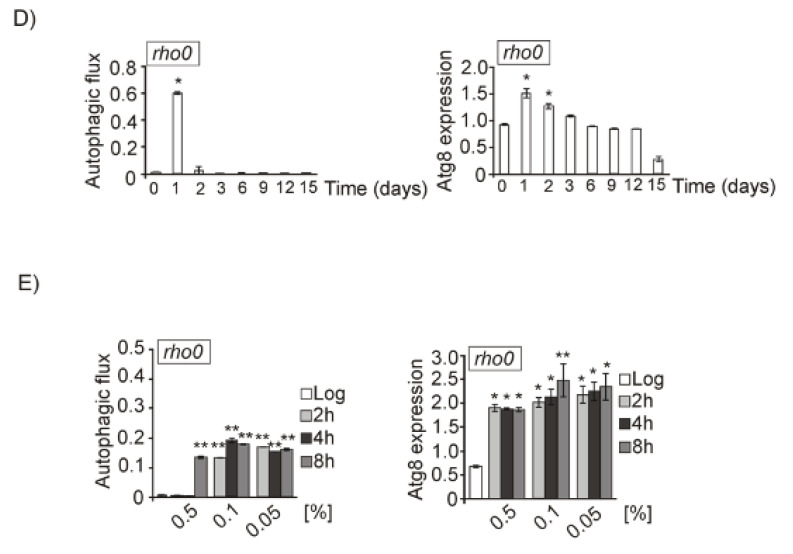
Mtl1 signals a decrease in glucose concentration to the autophagy machinery in a manner not fully dependent on ATP production by mitochondria. (**A**) wt and *mtl1* cells expressing GFP–Atg8 grown in SD media were transferred to minimum media devoid of glucose (0% glucose) to determine autophagic flux and Atg8 expression as in Figure 1B. (**B**) Upon transference to minimum media without glucose, ATP (at 200 mM final concentration) was added to the cultures described in (**A**) and samples were collected at the indicated times for determination of autophagy as in (**A**). (**C**) ATP (200 mM) was added to *mtl1* cultures growing in SD minimum medium for one day at the diauxic shift, and samples were collected at 4 and 8 h for autophagic flux and Atg8 expression determination, as in Figure 1B. (**D**) A *rho0* mutant expressing GFP–Atg8 was grown (as in Figure 1B) for autophagy determination. (**E**) *rho0* cells exponentially growing in SD were washed and transferred to several media containing different glucose concentrations to analyze autophagy, as in Figure 4A. Error bars in the histograms represent the standard deviation (SD) calculated from three independent experiments. Significance of the data was determinate by *p*-values from a Student’s unpaired *t*-test denoted as follows: * = 0.05 > *p* > 0.01; ** = 0.01 > *p* > 0.001.

However, placing cells at 0% glucose, we did not observe free GFP in either the Western blot or accumulated in vacuoles. Hence, bulk autophagy was not induced in wt or *mtl1* strains, as previously described by [67] (Figure 5A and Figure S4A). These authors attributed this result to the sudden lack of ATP required for the autophagy machinery. We added ATP to wt and *mtl1* cultures completely depleted of glucose and observed that whereas in wt cultures autophagy induction was high, in the *mtl1* mutant the autophagy response was only partially restored (Figure 5B and Figure S4B). Identical results were obtained during the diauxic shift in *mtl1* cultures when ATP was added to exponentially growing cells (Figure 5C and Figure S4C). These results led us to the conclusion that the absence of *MTL1* provoked ATP starvation when glucose concentration drops below a threshold. In the former experiment, we can observe that autophagy induction occurs when glucose concentration drops from 2% to 0.5% in wt cultures (Figure 4A and Figure S3A). However, in *mtl1* cultures, any decrease below 2% aborted autophagy (Figure 4C and Figure S3C).

In order to ascertain the contribution of mitochondrial ATP to autophagy response during the diauxic shift and a reduction in glucose concentrations in exponentially growing cultures, we analyzed a *rho0* mutant lacking mitochondrial DNA. We observed that the absence of mitochondrial DNA did not preclude the induction of bulk autophagy upon one day of culture (Figure 5D and Figure S4D) and upon glucose concentration reduction (Figure 5E and Figure S5E). These results suggest that the functional role that Mtl1 plays in the autophagy response to glucose availability is not only linked to ATP accumulation.

### 3.4. Both Ras2 or Sch9 Suppress mtl1 Deficiency in Bulk Autophagy Activation in All Metabolic Conditions That Imply Reduced Glucose Levels

We next tried to identify the pathway or pathways with which Mtl1 is connected to the signaling process converging on the autophagy machinery. In a previous study, we found that Mtl1 is negatively related to both Tor1 and Ras2 in response to oxidative stress and glucose starvation [53]. In addition, Mtl1 is also negatively related to Sch9, Slt2, and PKA during the diauxic shift or upon glucose depletion [30].

**Figure 6:**
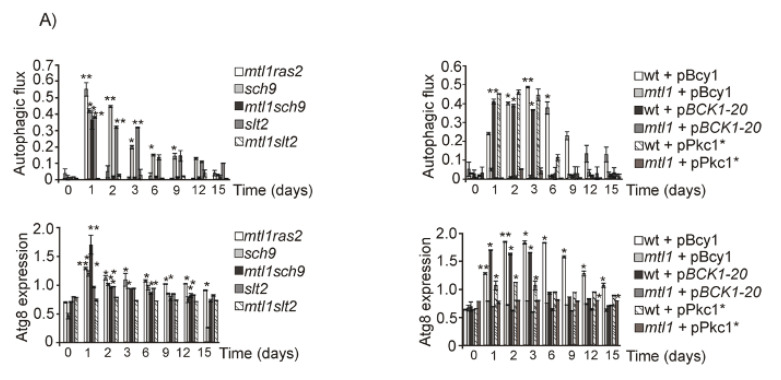

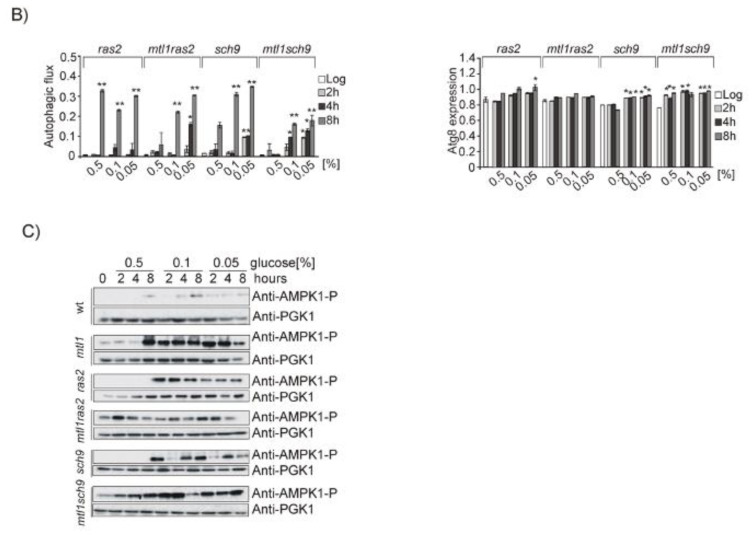
Both Ras2 and Sch9 suppress *mtl1* deficiency in autophagy signaling upon glucose concentration decreasing. (**A**) *mtl1ras2, sch9, mtl1sch9, slt2, mtl1slt2,* wt+pBcy1, *mtl1*+pBcy1, wt+p*BCK1-20*, *mtl1*+p*BCK1-20*, wt+pPkc1* and *mtl1*+pPkc1* strains expressing GFP–Atg8 were grown at 30 °C in SD media for 15 days. Samples were taken to determine autophagic flux and total Atg8 expression as described in Figure 1B. (**B**) Strains *ras2, mtl1ras2, sch9,* and *mtl1sch9* expressing GFP–Atg8 were exponentially grown in SD media to be subsequently transferred to minimum medium containing the indicated concentration of glucose. Samples were taken to determine autophagy as in Figure 4A. (**C**) wt samples (from Figure 4A) and *mtl1* samples (from Figure 4C) along with *mtl1, ras2, mtl1ras2, sch9* and *mtl1sch9* samples (from Figure 6B) were used for Western blot analysis and AMPK1 detection by using anti-AMPK1-P antibody. Error bars in the histograms represent the standard deviation (SD) calculated from three independent experiments. Significance of the data was determinate by *p*-values from a Student’s unpaired *t*-test denoted as follows: * = 0.05 > *p* > 0.01; ** = 0.01 > *p* > 0.001.

We demonstrated that Mtl1 mediates Bcy1 activating phosphorylation through TORC1 downregulation thus leading to PKA activation [30]. Therefore, and taking into consideration the hypothesis that *mtl1* mutants could cause impairment of the glucose signal through PKA, we also analyzed the overexpression of Bcy1, the PKA inhibitor, in both wt and *mtl1* strains. Overexpression of Bcy1 prolonged autophagy induction in wt cultures for longer times (Figure 6A and Figure S5A) as compared to the wt empty strain (Figure 1A and Figure S1A). However, Bcy1 overexpression did not restore autophagy in the *mtl1* mutant (Figure 3E, Figure 6A, Figures S2E and S5A). In previous papers, we observed clear impairment of Slt2 phosphorylation upon both oxidative stress and glucose deprivation in *mtl1* mutants [30]. Consistent with this information, we used a plasmid overexpressing the Pkc1 protein and a second plasmid bearing the *BCK1-20* allele which keeps Slt2 kinase constitutively activated, as Bck1 is the MAPKKK of the CWI pathway. Results depicted in Figure 3E, Figure 6A, Figures S2E and S5A demonstrate that the lack of bulk autophagy activation observed in the *mtl1* mutant, as a result of a decrease in glucose concentration during diauxic transition, is not caused by the lack of Slt2 kinase activity, since neither Pkc1 overexpression nor the *BCK1-20* allele restored the lack of autophagy induction in the *mtl1* mutant. We next decided to check Ras2 deletion in *mtl1*, since the GTPase is activated in the presence of glucose and is responsible for the synthesis of cAMP when glucose is the carbon source. Ras2 deletion in the *mtl1* mutant provoked the activation of bulk autophagy during the diauxic shift; in fact, the distribution levels of autophagy were equivalent between *ras2* and *mtl1ras2* (Figure 3B, Figure 6A, Figures S2B and S5A respectively) strains, suggesting that Mtl1 signals to Ras2 inactivation upon glucose starvation and diauxic transition signaling to activate bulk autophagy. Lastly, given the association between Mtl1 and Sch9 kinase [30], we also analyzed whether both proteins were also related to autophagy signaling. Deletion of *SCH9* restored autophagy in the *mtl1* mutant during the diauxic shift (Figure 6A and Figure S5A). This result suggests a connection of Mtl1 with Sch9 towards autophagy. In order to corroborate the glucose specificity of these responses, exponentially grown cultures of *mtl1ras2* and *mtl1sch9* along with the corresponding controls were assayed for bulk autophagy response upon glucose starvation (Figure 6B and Figure S5B). As expected, deletion of *RAS2* or *SCH9* suppressed the lack of autophagy induction in the absence of *MTL1*.

Snf1 is an AMPK family member which is highly conserved in eukaryotes. When glucose is exhausted at the beginning of the diauxic shift, Snf1 becomes activated to trigger a wide response of regulating activators and repressors to trigger respiratory metabolism (see review [21]). In order to detect a possible defect in Snf1 activation in the *mtl1* mutant, we analyzed Snf1 phosphorylation in Thr20 residues on the activation loop of the catalytic subunit in samples of wt*, mtl1, ras2, ras2mtl1, sch9* and *mtl1sch9*. In Figure 6C, it can be observed that Snf1 is correctly and similarly phosphorylated in all strains, concluding that *mtl1* defects in autophagy during the transition to stationary phase and upon glucose depletion are not a principal consequence of the lack of Snf1 activation as a response to glucose limitation.

In summary, our results suggest that either *RAS2* or *SCH9* deletion reverted the lack of autophagy in the *mtl1* mutant, suggesting that Mtl1 receives the signal of decreased glucose concentration and connects to both Ras2 and Sch9 inactivation, a mechanism that converges on macroautophagy induction. We have also observed similar results when the carbon source is either sucrose or fructose (Figure S2F).

### 3.5. Mtl1 Is Required for Mitochondrial Degradation Dependent on Atg33 and Independent of Atg32 during Chronological Ageing

**Figure 7:**
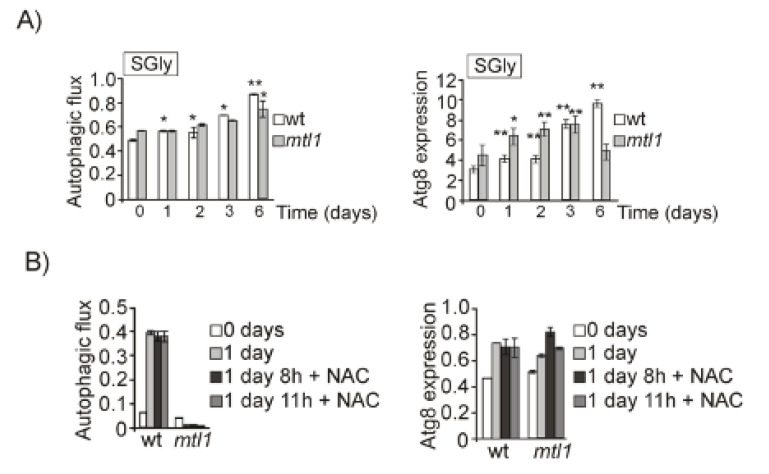
Mtl1 is not deficient in bulk autophagy in respiratory conditions. (**A**) wt and *mtl1* cultures were grown in minimum medium SGly (containing glycerol as unique carbon source) plus amino acids at 30 °C to stationary phase for 6 days. Samples were collected at the indicated times to identify macroautophagy as described in Figure 1B. (**B**) wt and *mtl1* cultures in SD medium growing to 1 day were treated with N-Acetyl cysteine (NAC) 5 mM for 8 and 11 h. Samples were collected for autophagy determinations as in (**A**). Error bars in the histograms represent the standard deviation (SD) calculated from three independent experiments. Significance of the data was determinate by *p*-values from a Student’s unpaired *t*-test denoted as follows: * = 0.05 > *p* > 0.01; ** = 0.01 > *p* > 0.001.

We wondered whether Mtl1 involvement in autophagy regulation would be related to any carbon source, not only to glucose availability. To answer this question, we decided to analyze a non-fermentative carbon source, glycerol, that forces cells to directly enter into respiratory metabolism. Mtl1 was clearly not involved in the detection of glycerol concentration linked to autophagy activity, since in both wt and *mtl1* cells we detected similar levels and patterns of autophagy (Figure 7A and Figure S6A). In a previous paper, we described that Mtl1 presented uncoupled respiration that provoked mitochondrial dysfunction and ROS accumulation [30]. In order to ascertain whether oxidative stress would be the cause of the autophagy problem, we added the antioxidant NAC to both wt and *mtl1* diauxic cultures (Figure 7B and Figure S6B). In order to demonstrate that NAC was exerting its antioxidant function, samples were collected and stained with dihydroethidium (DHE) for in vivo visualization of cellular oxidation in the fluorescent microscope (Figure S6C). Our results indicate that oxidative stress is not the cause of autophagy impairment during diauxic shift in the *mtl1* mutant (Figure 7B and Figure S6B,C).

**Figure 8:**
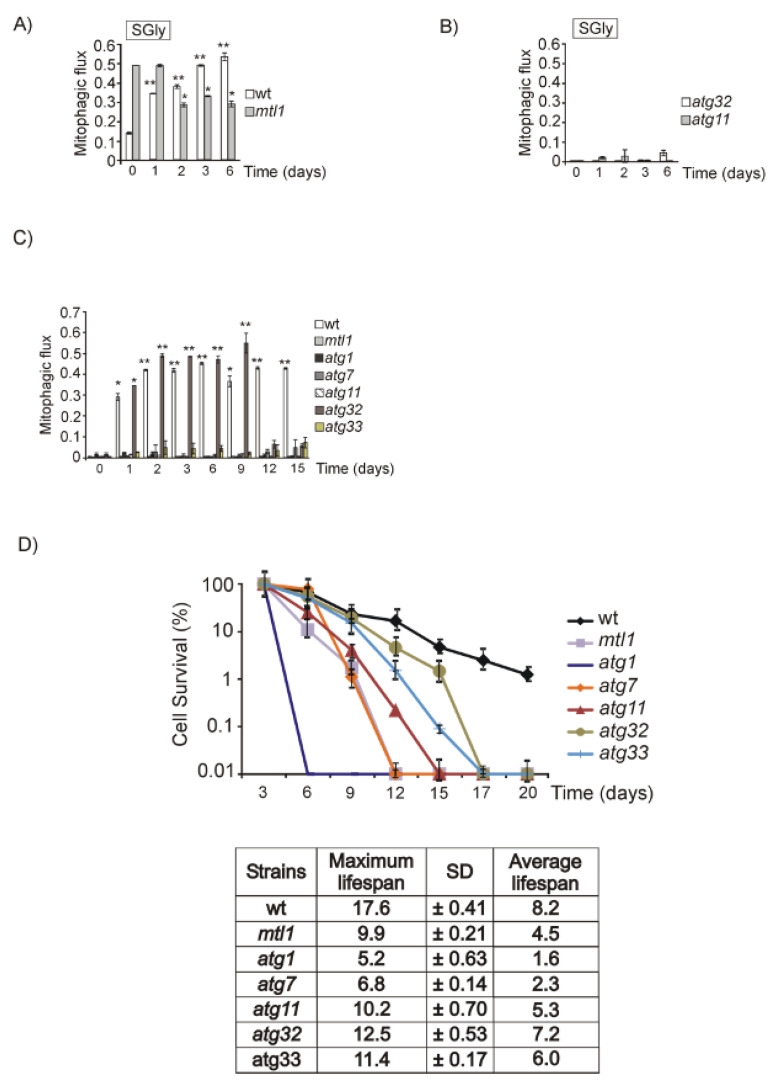

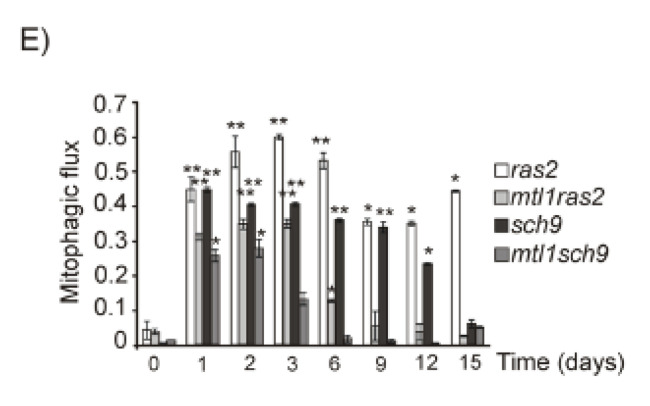
Mtl1 is needed for specific mitochondrial degradation during stationary phase. (**A**) wt and *mtl1* cultures transformed with the plasmid Idp1–GFP were grown in SGly media plus amino acids at 30 °C. Samples were taken at the indicated times to determine mitophagic flux. Mitophagic flux was calculated as the ratio between free GFP and total Idp1–GFP detected in the Western blot. (**B**) *atg32* and *atg11* cultures transformed with Idp1–GFP were grown in SGly medium plus amino acids at 30 °C. Samples were collected at the indicated times to determine mitophagic flux as in (**A**). (**C**) wt, *mtl1, atg1, atg7, atg11, atg32* and *atg33* strains bearing the plasmid Idp1–GFP were grown in SD media at 30 °C for 15 days in continuous shacking. Mitophagic flux was determined as in A. (**D**) Chronological life span curves for wt, *mtl1, atg1, atg7, atg11, atg32* and *atg33* strains cultured in SD media plus amino acids at 30 °C. Samples were taken at the indicated times to determine CLS, as described in the Materials and Methods. Numerical data regarding maximum life span (the day when cultures reach 10% survival) and average life span (the day at which 50% survival was recorded) for each strain is depicted. (**E**) *ras2, mtl1ras2, sch9* and *mtl1sch9* mutants, transformed with Idp1–GFP were treated as in (**C**) and mitophagic flux was determined as in (**A**). Error bars in the histograms represent the standard deviation (SD) calculated from three independent experiments. Significance of the data was determinate by *p*-values from a Student’s unpaired *t*-test denoted as follows: * = 0.05 > *p* > 0.01; ** = 0.01 > *p* > 0.001.

There is an alternative possibility, that if the problem of *mtl1* is mitochondrial function, we would expect to detect severe deficiencies in mitophagy. For this purpose, we analyzed mitophagy in cells expressing a fusion of mitochondrial matrix protein to GFP, Idp1–GFP, transformed in either wt or *mtl1* strains [68]. We monitored the vacuole clipping of the fusion protein Idp1–GFP. The identification of free GFP with anti-GFP antibody in a Western blot would reveal the existence of mitophagy, since GFP is very resistant to degradation [69]. Mitophagy studies are usually carried out in respiratory carbon sources or alternatively in stationary cultures. When wt and *mtl1* cells were grown in SGly to the stationary phase, we observed mitophagy in both strains (Figure 8A and Figure S7A) as opposed to *atg32* and *atg11* mutants, in which mitophagy was undetectable (Figure 8B and Figure S7B). Atg32 is a mitochondrial outer protein required to initiate mitophagy as a selective type of autophagy (see review [70]). Atg11 is a critical protein for selective autophagy; it is essential in selective and non-selective autophagy processes (see review [71]). From the results shown in Figure 8B and Figure S7B, we conclude that the *mtl1* mutant does not have any defects regarding mitophagy dependent on Atg32.

We also checked mitophagy during the diauxic shift and stationary phase in cultures grown in SD with glucose as the only carbon source. We observed mitochondrial degradation as free GFP derived from Idp1–GFP accumulated in vacuoles in wt cultures (Figure 8C and Figure S7C). However, this particular mitochondrial degradation was undetected in each of the *atg1*, *atg7* or *atg11* strains (Figure 8C and Figure S7C). This particular mitophagy-like activity was not dependent on Atg32, since we observed similar results in both wt and *atg32* strains (Figure 8C and Figure S7C). We discarded the possibility that our results reflected bulk autophagy, since *atg11* cultures did not demonstrate defects in bulk autophagy during the diauxic shift nor stationary phase (Figure 8C and Figure S7C). It has been reported in yeast that Atg33 is a mitophagy mitochondrial outer membrane protein [68] required for the stationary phase. We observed a deficiency in mitophagy when we analyzed the *atg33* mutant (Figure 8C and Figure S7C). More interesting was the finding that the *mtl1* mutant was as deficient as *atg11* and *atg33* in Idp1 mitophagy during the diauxic shift and stationary phase (Figure 8C and Figure S7C). The three mutants turned out to have shorter chronological life spans than the corresponding wt (Figure 8D). Our results suggest that yeast cultures in SD demonstrate mitochondrial degradation in the vacuole upon the diauxic shift and during the stationary phase through a selective autophagy process independent of Atg32 but dependent on the Atg1, Atg7, Atg11 and Atg33 proteins. We also demonstrate that Mtl1 plays a relevant role in initiating this mechanism; one potential target would be Atg33 that will have to be investigated further.

We decided to check whether the absence of mitochondrial degradation during the chronological life span of *mtl1* was also alleviated by either *RAS2* or *SCH9* deletion, and obtained equivalent results to those described for bulk autophagy, inactivation of *RAS2* or *SCH9* restored mitophagy-like degradation during the diauxic shift to *mtl1* mutants (Figure 8C,E and Figure S7C,D).

## 4. Discussion

Our results point to a situation by which gradual glucose depletion activates bulk autophagy, and for this response Mtl1 activity is essential. Unlike in wt cells, in the single mutant *mtl1* there was no a detectable response towards autophagy unless either *RAS2* or *SCH9* were deleted. Deletion of *RAS2* reverted the *mtl1* phenotype regarding bulk autophagy, which points to the importance of glucose availability and the switch from respiratory to fermentative metabolism, suggesting that during that transition the Ras2 pathway must be not active and Mtl1 is the connector between glucose and Ras2 activity. This is supported by the observation that these results also extended to other fermentative sugars (Figure S2F). Interestingly, the signal to autophagy in the models of glucose deprivation did not flow to TORC1, nor to the PKC1 pathway or PKA, but directly to the autophagy machinery to phosphorylate the Atg1 protein. Accordingly, some authors [2] already observed that TORC1 does not seem to play a principal function in glucose starvation.

According to former studies [67], the abrupt transition from 2% glucose to 0% glucose does not activate macroautophagy, because for this mechanism, ATP is essential and the mentioned transition causes cells to be suddenly exhausted for ATP. This is understandable, as cells are transferred from a culture containing high glucose concentrations to a culture without glucose nor other carbon source. However, during the transition from fermentative to respiratory metabolism, the decrease in glucose concentration occurs gradually. In wt cells, autophagy was activated already when the glucose concentration reached a value of 0.5% (27.77 mM), and reached maximum values when glucose levels decreased to 0.05% (0.13 mM), whereas in *mtl1* mutant autophagy was never induced. We hypothesized that this could have occurred because there might be a threshold for cells to sense glucose levels (or other alternative fermentable sugars), which would activate autophagy to obtain energy and nutrients probably linked to the induction of respiratory metabolism. Mtl1 could be the sensor for this threshold, and if unable to switch properly could consequently be unable to induce autophagy. In a previous study we observed that the *mtl1* mutant accumulates higher cAMP levels than wt cells. We believe that since the *mtl1* mutant has high cyclic AMP accumulation both in exponential or stationary cultures [28], cells would be depleted of ATP, with consequent Ras2 deletion potentially compensating for that depletion, thus avoiding the accumulation of cyclic AMP in *mtl1*. Nonetheless, this hypothesis was not sustained, since other nutrient stresses were capable of activating bulk autophagy in *mtl1* exponential cells (Figure 4C). It could be argued that during the diauxic shift, glucose reduction forces the switch from fermentative to respiratory metabolism and in these circumstances the main ATP source would be mitochondrial. Since in the absence of Mtl1 the ratio of cAMP/ATP would be higher than in wt cells, this would generate a signal of glucose starvation leading to the blockade of autophagy. However, our results demonstrate that the absence of mitochondrial DNA, and consequently the absence of ATP production through respiration metabolism (*rho0* mutant) did not preclude autophagy induction in glucose reduction conditions. Consequently, we also discarded this second hypothesis.

In humans, ATP increases autophagic flux in some cell lines but not in others [72]. Moreover, the addition of ATP to *mtl1* cultures only partly restored at a low degree of autophagy both in the diauxic transition and upon partial glucose depletion. This supports the model by which Mtl1 function couples glucose starvation to Ras activity and autophagy induction.

In agreement with the studies of [67], we observed that after full reduction of glucose, no nutritional stress could provoke bulk autophagy induction (rapamycin, amino acids, nitrogen, or iron).

In this study, we also present a nutritional model by which Gcn2 detects the signal of amino acid deprivation connecting to bulk autophagy in a manner independent of TORC1 activity. Hence, our data demonstrates that both Mtl1 and Gcn2 are key factors that induce bulk autophagy during the starvation process involving firstly glucose and secondly amino acid deprivation that occurs during the transition from fermentative to respiratory metabolism.

The observation that the *mtl1* mutant grows in the presence of glycerol as the only carbon source at similar rates to wt cells, indicates that mitochondrial function is sufficient to support the ATP requirements in this condition. Moreover, we also observed that Mtl1 did not participate in the induction of mitophagy in glycerol cultures dependent on Atg32 (Figure 8A,B).

We observed that either Mtl1 or Atg11 (involved in selective autophagy), are required to degrade mitochondria during transition to the stationary phase in synthetic media containing glucose as the only carbon source. This degradation process depends on the autophagy machinery, since in the absence of Atg7 or Atg1 it does not take place. Our data are consistent with a previous paper in which the authors showed that autophagy in response to carbon starvation requires Atg11 as a scaffold protein for the PAS [67]. Nevertheless, the mitophagy-like process that occurred in media containing glucose is independent of Atg32 but dependent on Atg33 (Figure 8C). This is not unexpected, since Atg33 was characterized as an autophagy protein specific to *Saccharomyces cerevisiae* whose role was linked to the induction of mitophagy during the stationary phase [68]. Whether or not the mitochondrial degradation that we observed during the stationary phase is a type of mitophagy dependent of Atg11 and Atg33 but independent of Atg32, should be further analyzed in future studies. Consequently, the availability of glucose as a carbon source has specific responses regarding autophagy in which Mtl1 is directly involved.

Snf1 is an AMPK orthologous to the mammalian AMP-kinase (reviewed in [73]) whose activity has been reported to be required in response to glucose starvation [21,74] to downregulate autophagy in the stationary phase [23], and is also required to induce autophagy in that context [67]. Snf1p is a catabolic regulator that is activated by an increase in the ADP/ATP ratio [75]. Nonetheless, in our studies Snf1, both in *mtl1* mutant or in wt cells, is highly activated during the diauxic shift when glucose levels are reduced and this activity is high during the stationary phase, therefore we cannot attribute lack of Snf1 activity to the defects observed in the *mtl1* mutant.

The conclusion to our data is that the transition from high to low concentrations of glucose triggers the connection between Mtl1 and autophagy and is not fully dependent on mitochondrial function and ATP accumulation. Another argument to support our hypothesis is that in the *mtl1* mutant, the presence of PAS is clearly detectable in the diauxic transition and in the presence of low glucose concentrations (Figure 3F), as opposed to that observed by previous authors [67] in the absence of ATP.

The linkage between Mtl1 and Ras2 has been previously described [28]. Our data suggest that Ras2 is the key regulator of bulk autophagy and the autophagy of mitochondria during the stationary phase. Whether or not this connection is related to mitochondria is at this moment unknown, since direct evidence for a regulation of mitochondria by Ras via cAMP–PKA is absent. Moreover, it is not unlikely that the Ras protein acts independently of adenylate cyclase and cAMP according to [76]. PKA is one of the effectors of Ras2 [77,78], and is also involved in autophagy regulation. However, concerning Mtl1 signaling in autophagy, PKA does not appear to be required as an intermediary molecule. Moreover, constitutive activation of the RAS-cAMP signaling pathway confers resistance to rapamycin [79,80,81]. This would explain why in an *mtl1* mutant, rapamycin does not provoke the induction of autophagy in the diauxic shift (not shown), taking into consideration the hypothesis that Mtl1 helps in the switch from fermentative to respiratory metabolism through the Ras/cAMP pathway.

Sch9 is a kinase effector of the TORC1 pathway [82]. In addition, Sch9 has been described as acting in a different pathway than PKA in glucose response. This is in agreement with our observations that Bcy1 overexpression or *SLT2* deletion did not suppress the lack of autophagy observed in the *mtl1* mutant. Our results are in line with the conclusion that TORC1, PKA, and Sch9 independently regulate autophagy during growth [17,83]. Deletion of Sch9 is sufficient to activate autophagy [17]. Here, we observed that the *sch9* mutant suppressed the lack of both bulk autophagy and mitophagy of *mtl1* during the diauxic shift and in conditions of low glucose, restricting the signal of glucose availability. Sch9 is also implicated in the selective autophagic degradation of ribosomes, mitochondria, and peroxisomes [84,85]. Although we still do not have a certain interpretation of the fact that Sch9 regulates ribosomal gene transcription, and that ribosomal biogenesis is one of the mechanisms requiring more ATP consumption, one interpretation would be that diminishing the level of ribosomal biogenesis also diminishes ATP consumption, perhaps favoring the induction of autophagy. The molecular mechanisms underlying the involvement of cAMP in the induction of autophagy related to nutritional starvation conditions deserves future investigation.

Our findings suggest that the Mtl1 cell wall receptor of the CWI pathway is a glucose sensor required to activate both bulk autophagy and Atg33-Atg11 mitophagy in response to glucose concentration decreases. Activation occurs through either Ras2 or Sch9 inactivation converging on Atg1 phosphorylation.

## Figures and Tables

**Table 1:** Yeast strains.

Strain	Genotype	Source
CML128	*MATa leu2D3,112, ura3D52, trp1D0, his4D0*	[52]
GSL011	*MATa mtl1::NatMx4*	[53]
GSL053	*MATa ras2::Leu2MX5*	[53]
GSL054	*MATa mtl1::KanMx4 ras2::LEU2Mx5*	[53]
GSL197	*MATa leu2D3,112, ura3D52, trp1D0, his4D0 URA3::GFP-ATG8*	[23]
GSL198	*MATa mtl1::KanMx4 URA3::GFP-ATG8*	This work
GSL199	*MATa tor1::KanMx4 URA3::GFP-ATG8*	[23]
GSL200	*MATa mtl1::KanMx4 tor1::LEU2 URA3::GFP-ATG8*	This work
GSL201	*MATa ras2::Leu2MX5 URA3::GFP-ATG8*	[23]
GSL202	*MATa mtl1::KanMx4 ras2::LEU2 URA3::GFP-ATG8*	This work
GSL205	*MATa sch9::NatMx4*	[30]
GSL206	*MATa sch9::NatMx4 mtl1::KanMx4*	[30]
GSL218	*MATa atg7::NatMx4*	[23]
GSL226	*MATa atg7::NatMx4 URA3::GFP-ATG8*	[23]
GSL265	*MATa slt2::NatMx4 URA3::GFP-ATG8*	This work
GSL279	*MATa sch9::NatMx4 URA3::GFP-ATG8*	This work
GSL293	*MATa atg11::NatMx4*	[23]
GSL296	*MATa atg33::NatMX4*	This work
GSL297	*MATa atg11::NatMx4 URA3::GFP-ATG8*	[23]
GSL324	*MATa atg1::NatMx4*	[23]
GSL352	*MATa gcn2::KanMx4 URA3::GFP-ATG8*	[23]
GSL364	*MATa atg32::KanMx4*	[23]
GSL370	*MATa rho0 URA3::GFP-ATG8*	[23]
GSL372	*MATa leu2D3,112, uras3D52, trp1D0, his4D0 ATG1-HA::LEU2*	[23]
GSL382	*MATa snf1:KanMx4 URA3::GFP-ATG8*	[23]
GSL414	*MATa mtl1::KanMX4 sch9::NatMx4 URA3::GFP-ATG8*	This work
GSL415	*MATa mtl1::KanMx4 slt2::NatMx4 URA3::GFP-ATG8*	This work
GSL416	*MATa mtl1:NatMx4 ATG1-HA::LEU2*	This work
BY4741 pho8Δ	*MATa pho8 his3D1, leu2D0, met15D0, ura3D0*	[54]

**Table 2:** Plasmids employed.

Plasmid	Restriction Sites to Clone the ORF	Marker	Promoter	Epitope	Source
pGFP-Atg8	EcoRI, XhoI	*URA3*	ATG8	GFP	[55]
pSfp1-GFP	SalI, SmaI	*URA3*	MET25	GFP	[23]
pAdh1-Msn2-GFP	KspI, SalI	*LEU2*	ADH1	GFP	[56]
pRtg1-GFP	XhoI, EcoRI	*URA3*	RTG1	GFP	[57]
pIdp1-GFP	HindIII, XhoI	*URA3*	IDP1	GFP	[58]
pAtg13-HA	NotI, PstI	*URA3*	ADH1	HA	[23]
pAtg1-HA	NotI, PstI	*LEU2*	ADH1	HA	[23]
pMM351	PstI, HindIII	*LEU2*	ADH1	HA	[51]
pBcy1HA	SmaI, XhoI	*HIS3*	ADH1	HA	[30]
pPkc1*	PmeI, NotI	*LEU2*	ADH1	HA	This work
p*BCK1-20*	HindIII, PstI	*TRP1*	LAC		[28]
pYX242-cytPho8	AvrII, MluI	*LEU2*	PHO8		[54]

## Data Availability

Not applicable.

## Supplementary Materials

The following are available online at https://www.mdpi.com/article/10.3390/jof7110903/s1, Figure S1. Sequential descent of glucose and amino acids activates bulk autophagy during the diauxic shift in *Saccharomyces cerevisiae*. Figure S2. Mtl1 and Gcn2 control autophagy induction during glucose and amino acid starvation. Figure S3. Mtl1 signals glucose limitation to the autophagy machinery. Figure S4. Mtl1 signals decreases in glucose concentration to the autophagy machinery in a manner not fully dependent on ATP production by mitochondria. Figure S5. Both Ras2 and Sch9 suppress *mtl1* deficiency in autophagy signaling upon glucose concentration descent. Figure S6. Mtl1 is not deficient in bulk autophagy in respiratory conditions. Figure S7. Mtl1 is needed for specific mitochondrial degradation during stationary phase.
