# Supplementary material for: The Cell Wall Integrity Receptor Mtl1 Contributes to Articulate Autophagic Responses When Glucose Availability Is Compromised
Source: J Fungi (Basel). 2021 Oct 26;7(11):903. doi: 10.3390/jof7110903 (PMC8623553; doi:10.3390/jof7110903)
Supplement: Supplementary file 1 [file jof-07-00903-s001.zip › jof-1414793-supplementary.pdf]

Figure S1

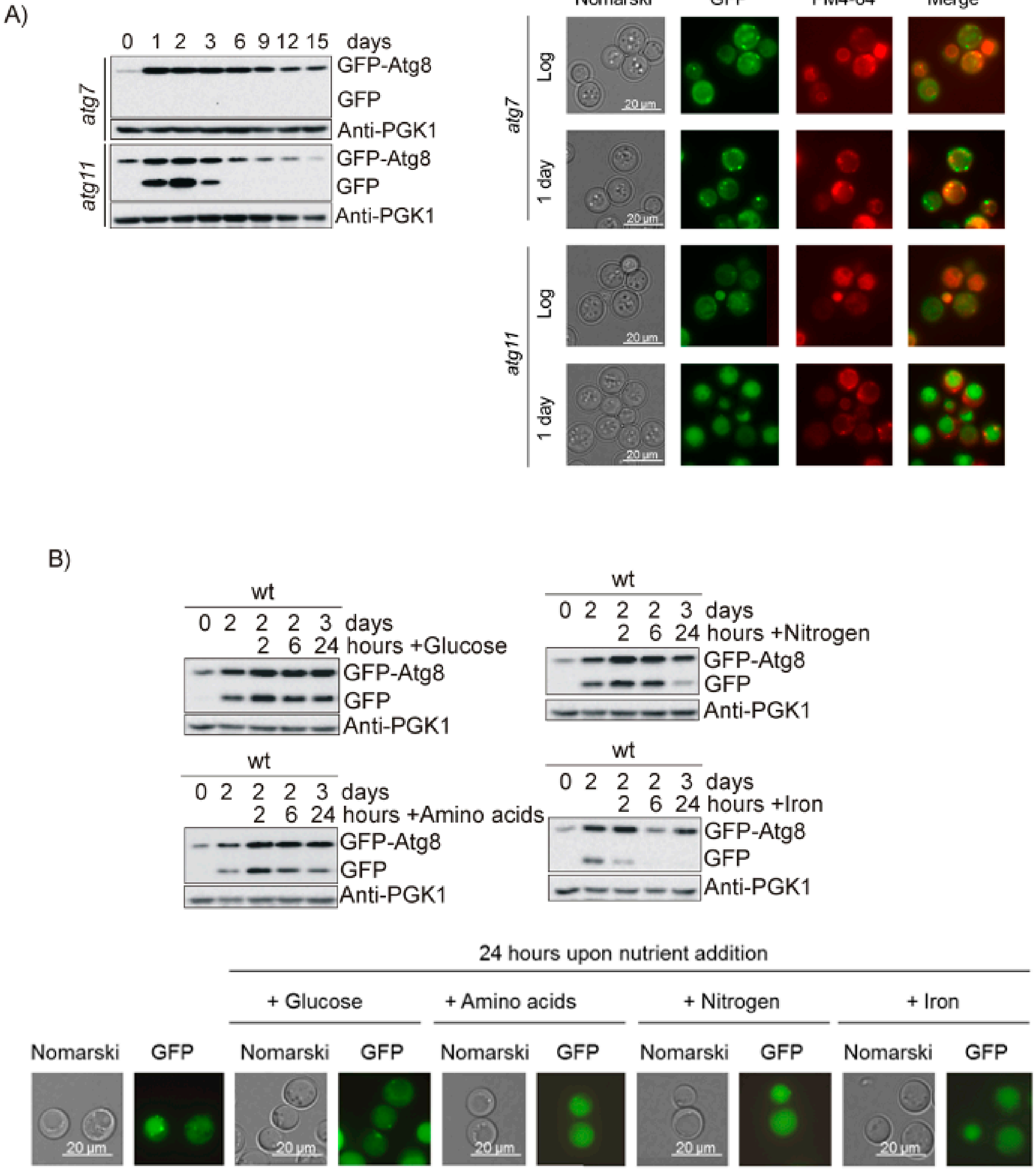
A)
0 1 2 3 6 9 12 15 days
atg7
GFP-Atg8
GFP
Anti-PGK1
atg11
GFP-Atg8
GFP
Anti-PGK1
Nomarski
GFP
FM4-64
Merge
Log
1 day
20 µm
B)
wt
0 2 2 2 3 days
2 6 24 hours +Glucose
2 6 24 hours +Nitrogen
2 6 24 hours +Amino acids
2 6 24 hours +Iron
GFP-Atg8
GFP
Anti-PGK1
24 hours upon nutrient addition
+ Glucose
+ Amino acids
+ Nitrogen
+ Iron
Nomarski
GFP

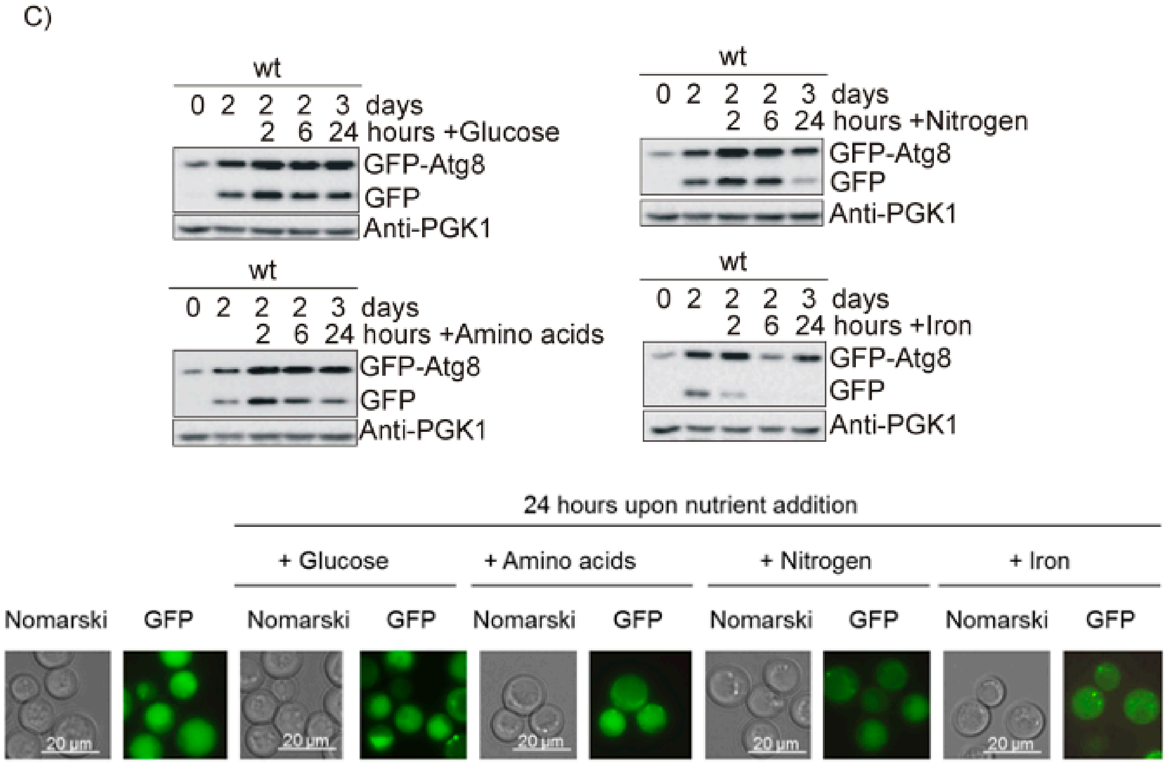

Figure S1. Sequential descent of glucose and amino acids activates bulk autophagy during the diauxic shift in *Saccharomyces cerevisiae*. A) *atg7* and *atg11* strains expressing the fusion protein GFP-Atg8, were grown to log phase (OD_{600}: 0.6) in SD medium at 30ºC. Aliquots were collected for total protein extraction, western blot and for *in vivo* observation of GFP-Atg8 in the fluorescence microscope as in A. B) wt cells bearing GFP-Atg8 in the genome was exponentially grown at OD_{600}: 0.6 at 30ºC in SD media and a sample was collected for analysis. Upon one day of culture, 2% glucose, amino acids (60 mg/ml Leucine, 20 mg/ml Histidine and 20 mg/ml Tryptophan), 0.67% nitrogen or 10 mM iron, were respectively added to the cultures and samples were collected upon 2, 6 and 24 hours to detect GFP-Atg8 by western blot and *in vivo* cellular localization through the fluorescence microscope as in A. Microscopic images represent GFP-Atg8 intracellular localization 24 hours upon re-feeding with each specific nutrient. C) As in D but results correspond to two days of growth.

Figure S2

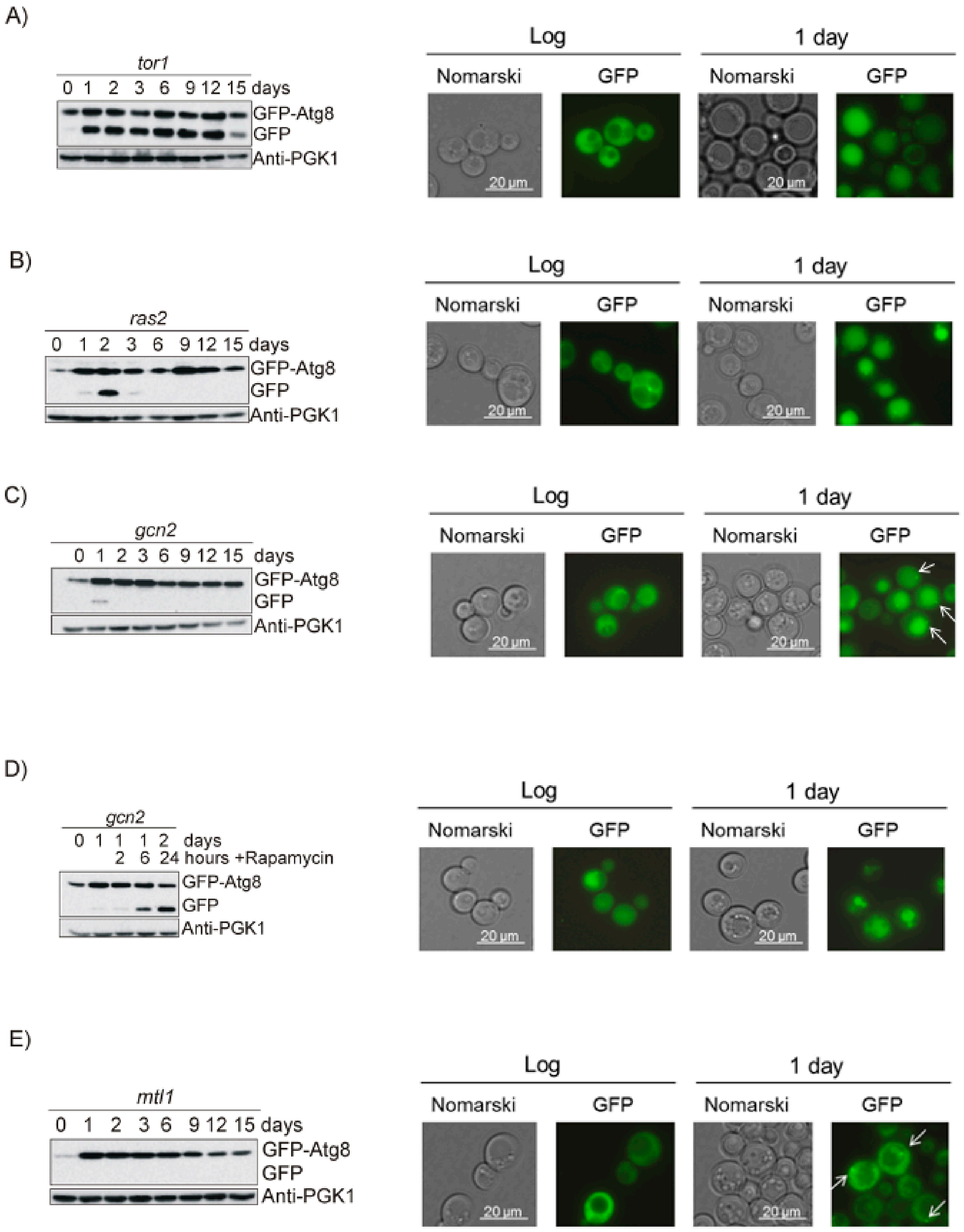

A)
tor1
0 1 2 3 6 9 12 15 days
GFP-Atg8
GFP
Anti-PGK1
Log
1 day
Nomarski
GFP
Nomarski
GFP
20 μm
20 μm
B)
ras2
0 1 2 3 6 9 12 15 days
GFP-Atg8
GFP
Anti-PGK1
Log
1 day
Nomarski
GFP
Nomarski
GFP
20 μm
20 μm
C)
gcn2
0 1 2 3 6 9 12 15 days
GFP-Atg8
GFP
Anti-PGK1
Log
1 day
Nomarski
GFP
Nomarski
GFP
20 μm
20 μm
D)
gcn2
0 1 1 1 2 days
2 6 24 hours +Rapamycin
GFP-Atg8
GFP
Anti-PGK1
Log
1 day
Nomarski
GFP
Nomarski
GFP
20 μm
20 μm
E)
mtl1
0 1 2 3 6 9 12 15 days
GFP-Atg8
GFP
Anti-PGK1
Log
1 day
Nomarski
GFP
Nomarski
GFP
20 μm
20 μm

F)

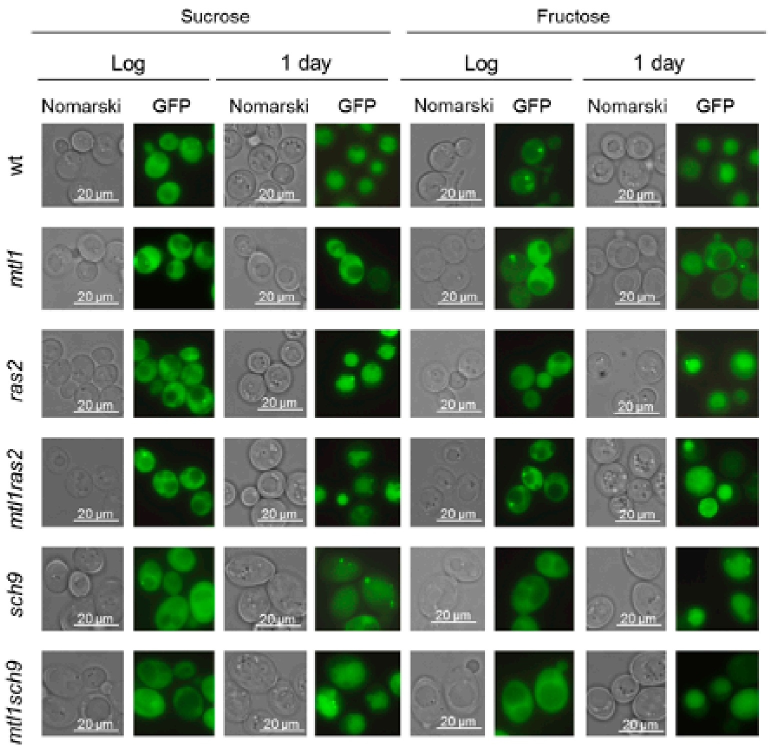

Figure S2. Mtl1 and Gcn2 control autophagy induction during glucose and amino acids starvation. Growth conditions, western blot and in vivo microscope observation determined in: A) *tor1* mutant expressing GFP-Atg8; B) *ras2* mutant expressing GFP-Atg8 and C) *gcn2* mutant expressing GFP-Atg8, was performed as described in (Figure S1A). D) *gcn2* bearing GFP-Atg8 was exponentially grown at 30ºC in SD plus amino acids. Rapamycin (200 ng/ml) was added to the cultures upon 1 day of growth and samples were subsequently collected upon 2, 6 and 24 hours of exposure to the drug. Aliquots were treated as in (Figure S1A). E) Growth conditions, GFP-Atg8 *in vivo* intracellular localization and western blot in *mtl1* culture expressing the fusion protein GFP-Atg8 was performed as in (Figure S1A). F) wt, *mtl1*, *ras2*, *mtl1ras2*, *sch9* and *mtl1sch9* cultures in which the GFP-Atg8 fusion protein was integrated, were grown to log phase and 1 day in Sucrose or Fructose medium at 30º. Aliquots were collected for in vivo microscopic observation by using a fluorescence microscope.

Figure S3

A)

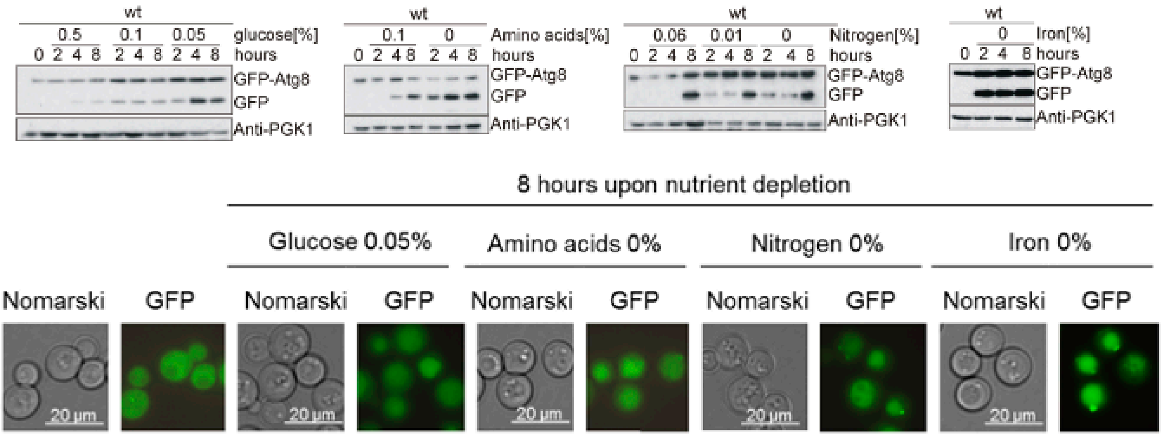

B)

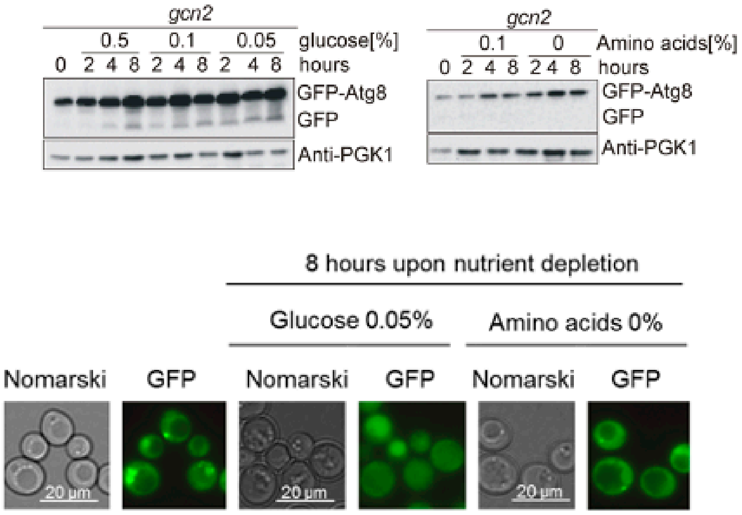

C)

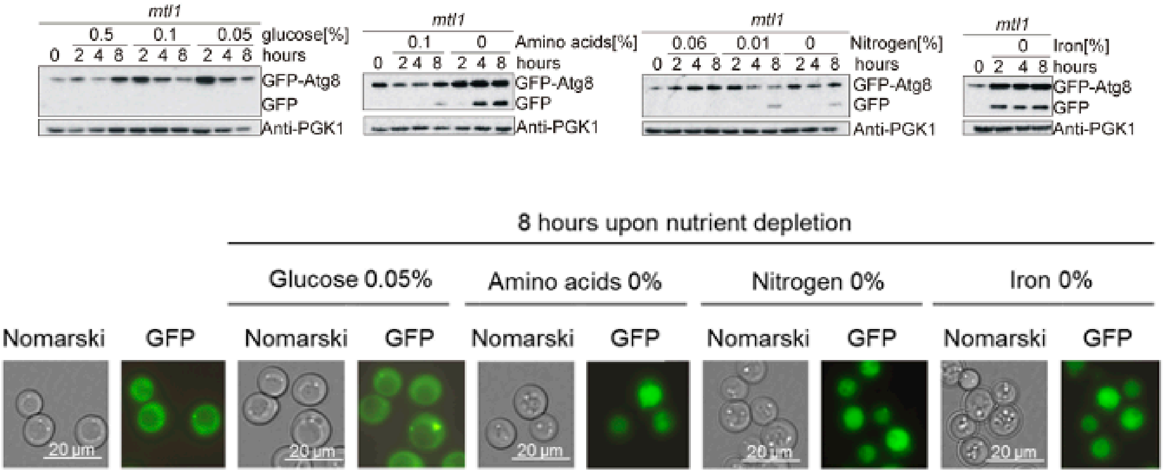

Figure S3. Mtl1 signals glucose limitation to the autophagy machinery. A) wt cells expressing GFP-Atg8 were exponentially grown in SD media. Aliquots were taken,

washed and transferred to different minimum media containing: 0.5, 0.1 or 0.05% glucose; 0.1 or 0% amino acids; 0.06, 0.01 or 0% nitrogen or medium without iron (0%). Autophagy was determined upon western blot analysis or *in vivo* identification of GFP-Atg8 in the fluorescence microscope as in (Figure S1A). The same experiments as in A were carried out in B) *gcn2* mutant cultures expressing GFP-Atg8 and in C) *mtl1* strain expressing GFP-Atg8.

Figure S4

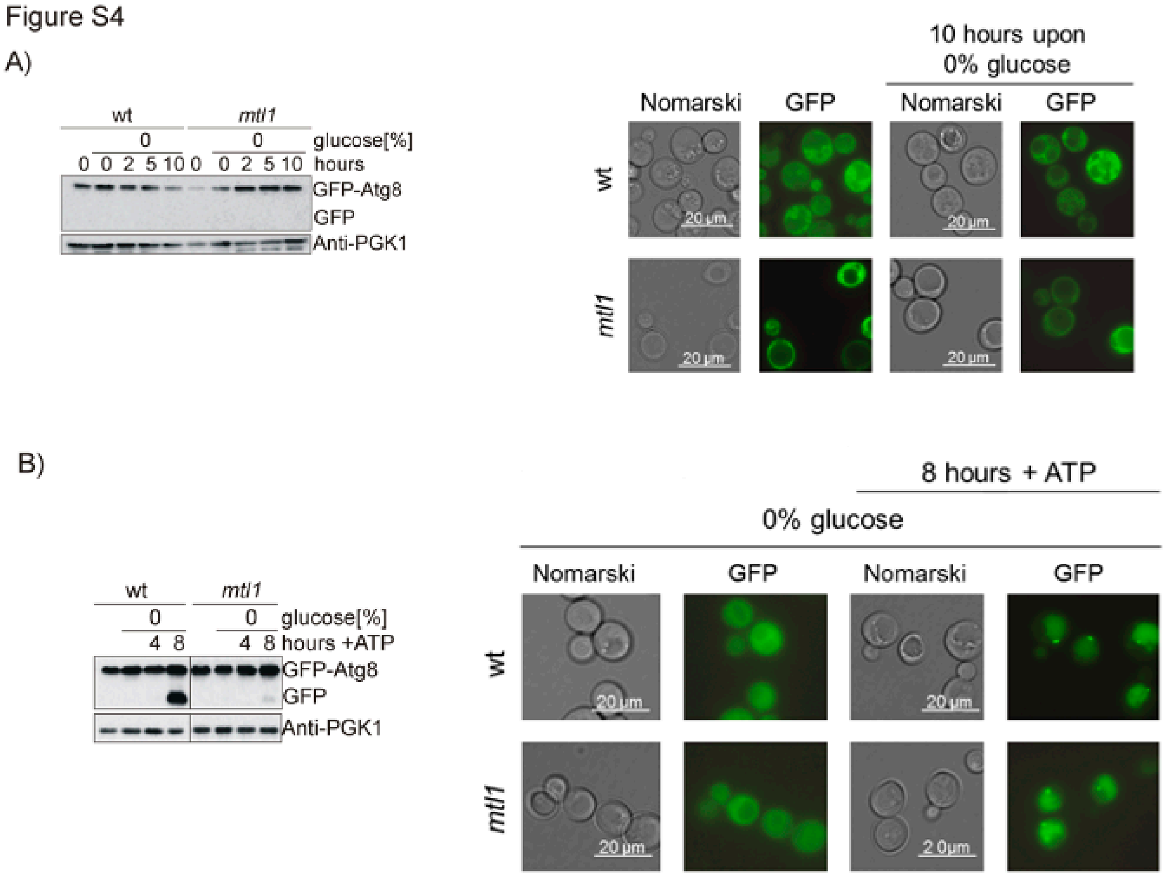
A)
wt
mtl1
0
glucose[%]
0 0 2 5 10 0 0 2 5 10 hours
GFP-Atg8
GFP
Anti-PGK1
10 hours upon
0% glucose
Nomarski
GFP
Nomarski
GFP
wt
mtl1
20 µm
B)
8 hours + ATP
0% glucose
wt
mtl1
0
0
glucose[%]
4 8
4 8
hours +ATP
GFP-Atg8
GFP
Anti-PGK1
Nomarski
GFP
Nomarski
GFP
20 µm
2 0µm

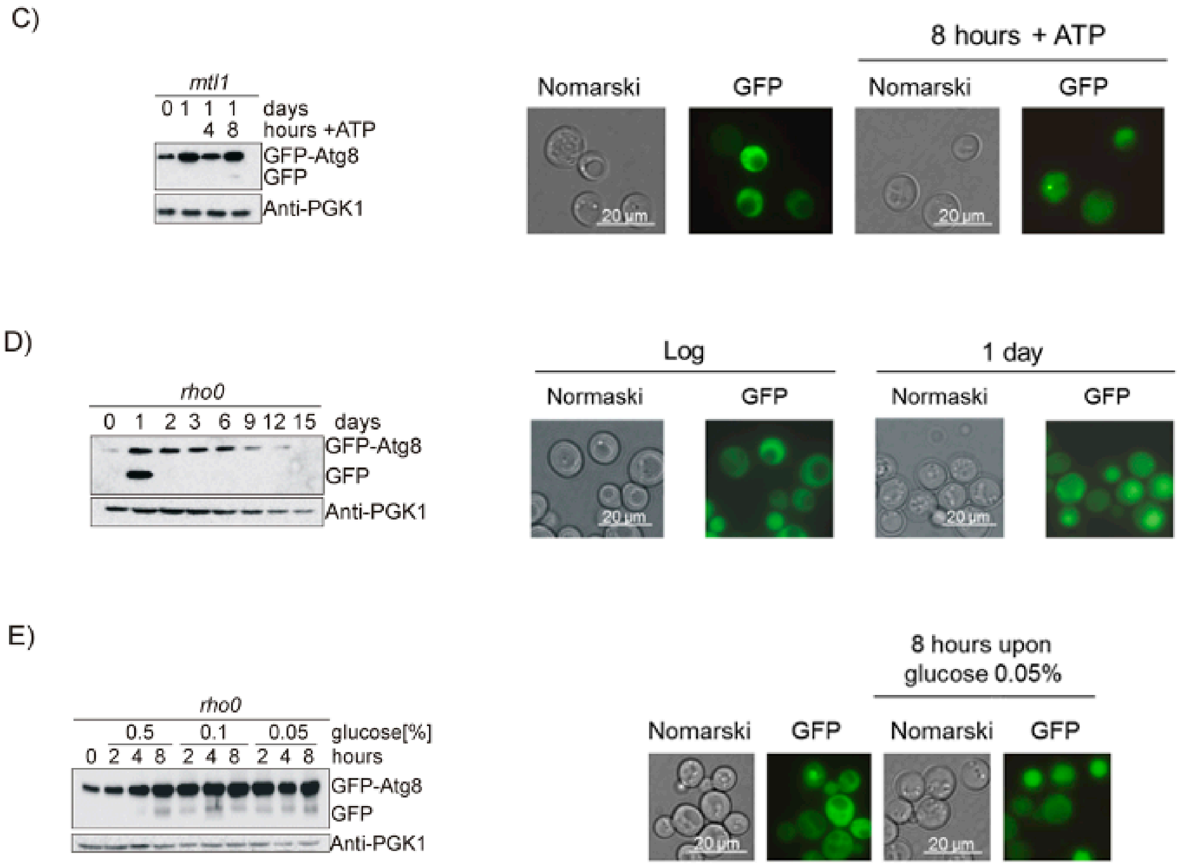

Figure S4. Mtl1 signals the decrease in glucose concentration to the autophagy machinery in a manner not fully dependent on ATP production by mitochondria. A) wt and *mtl1* cells expressing GFP-Atg8 grown in SD media were transferred to minimum media devoid of glucose (0% glucose) to determine autophagy upon detection of GFP-Atg8 vacuolar cleavage by western blot analyses and also *in vivo* through fluorescence microscopy as in (Figure S1A). B) Upon transference to minimum media without glucose, ATP (at 200 mM final concentration) was added to the cultures described in A and samples were collected at the indicated times for western blot and microscopic determination of autophagy as in A. C) ATP (200 mM) was added to *mtl1* cultures growing in SD minimum medium for one day, at the diauxic shift, and samples were collected at 4 and 8 hours for western blot analysis and *in vivo* observation in the fluorescence microscope. D) *rho0* mutant expressing GFP-Atg8 was grown as in (Figure S1A) for autophagy determination. E) *rho0* cells exponentially growing in SD were washed and transferred to several media containing different glucose concentrations to analyse autophagy as in (Figure S3A).

Figure S5

A)

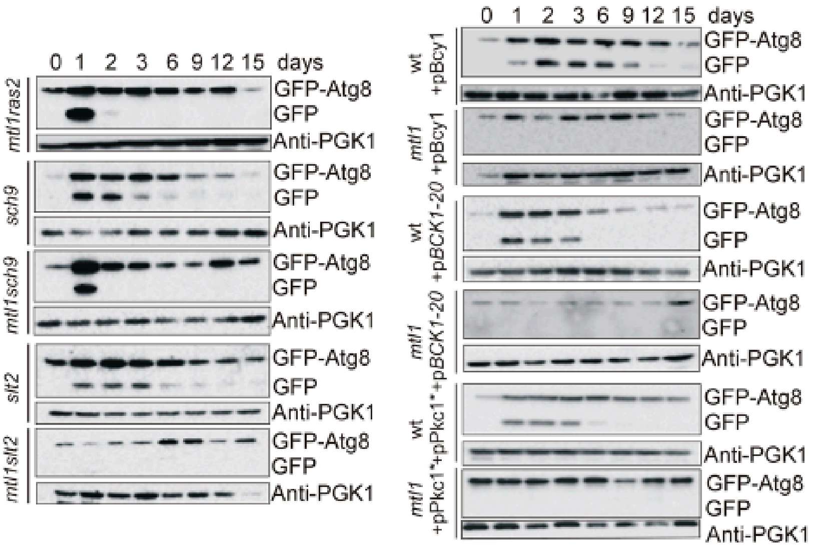
0 1 2 3 6 9 12 15 days
mtl1ras2
sch9
mtl1sch9
slt2
mtl1slt2
wt +pBcy1
mtl1 +pBcy1
wt +pBCK1-20
mtl1 +pBCK1-20
wt +pPkc1*
mtl1 +pPkc1*
GFP-Atg8
GFP
Anti-PGK1

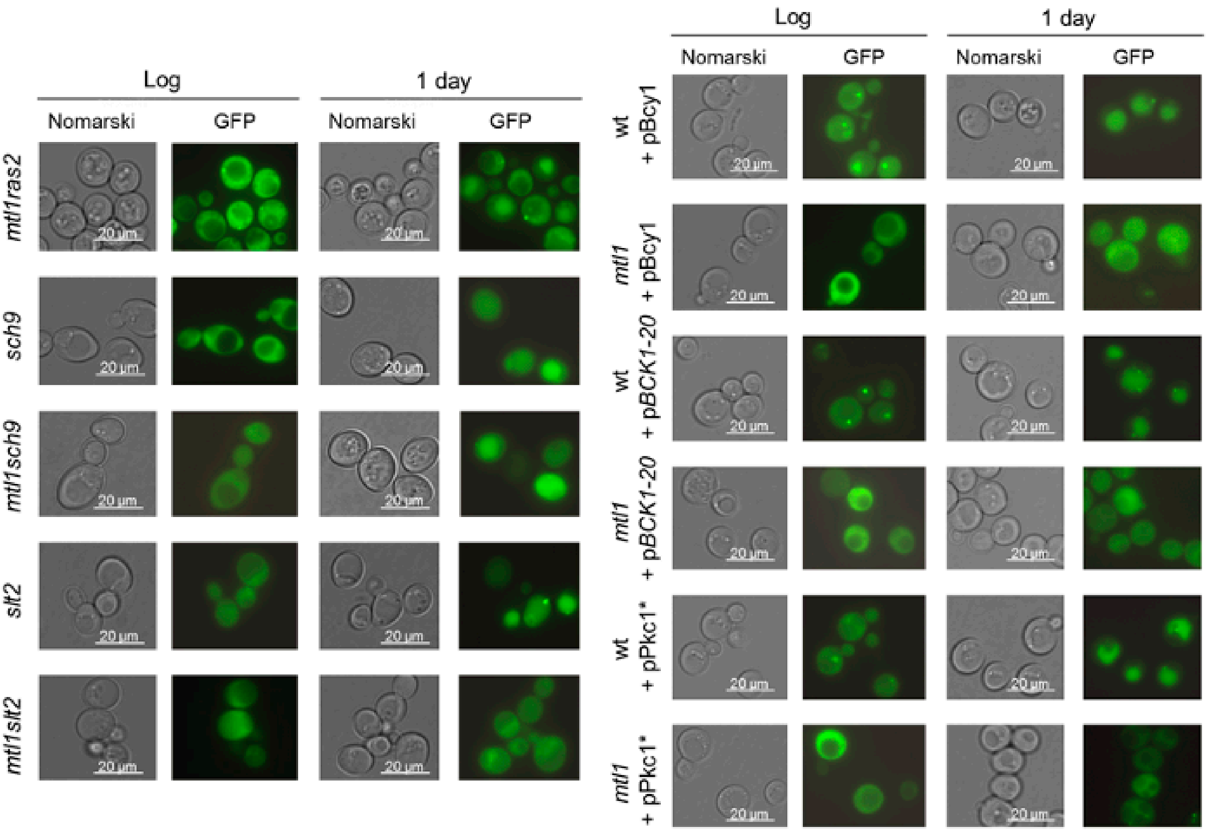
Log
1 day
Nomarski
GFP
mtl1ras2
sch9
mtl1sch9
slt2
mtl1slt2
wt + pBcy1
mtl1 + pBcy1
wt + pBCK1-20
mtl1 + pBCK1-20
wt + pPkc1*
mtl1 + pPkc1*
20 µm

B)

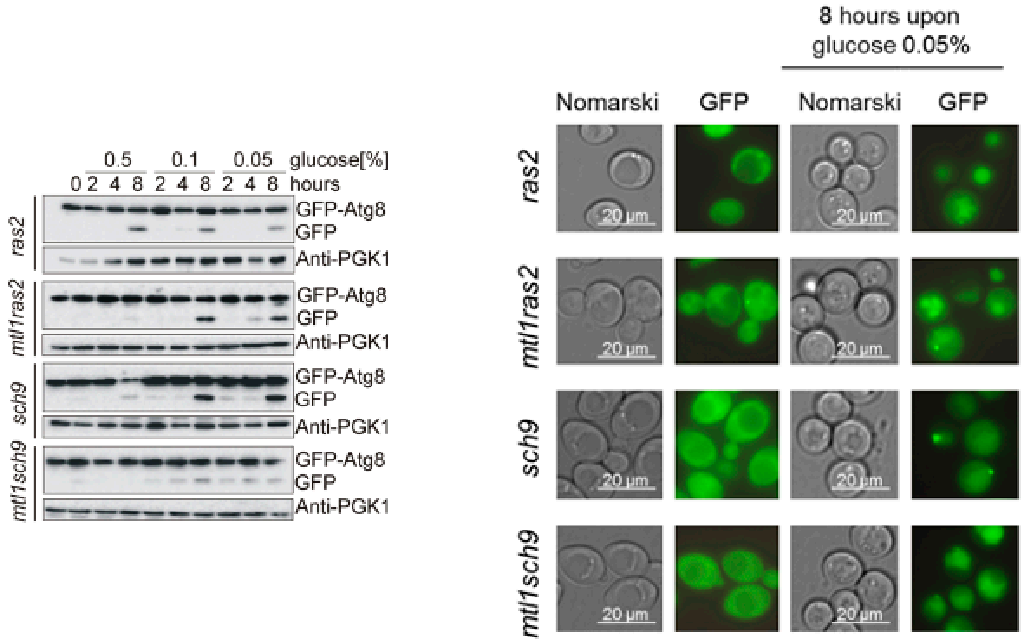

Figure S5. Both Ras2 and Sch9 suppress *mtl1* deficiency in autophagy signaling upon glucose concentration descent. A) *mtl1ras2, sch9, mtl1sch9, slt2, mtl1slt2,* wt+pBcy1, *mtl1*+pBcy1, wt+p*BCK1-20*, *mtl1*+p*BCK1-20*, wt+pPkc1* and *mtl1*+pPkc1* strains expressing GFP-Atg8 were grown at 30ºC in SD media during 15 days. Samples were taken for western blot analysis and *in vivo* observation in the fluorescence microscope of GFP cleavage from GFP-Atg8 were all performed as described in (Figure S1A). B) Strains *ras2, mtl1ras2, sch9* and *mtl1sch9* expressing GFP-Atg8 were exponentially grown in SD media to be subsequently transferred to minimum medium containing the indicated concentrations of glucose. Samples were taken to determine autophagy as in (Figure S3A).

Figure S6

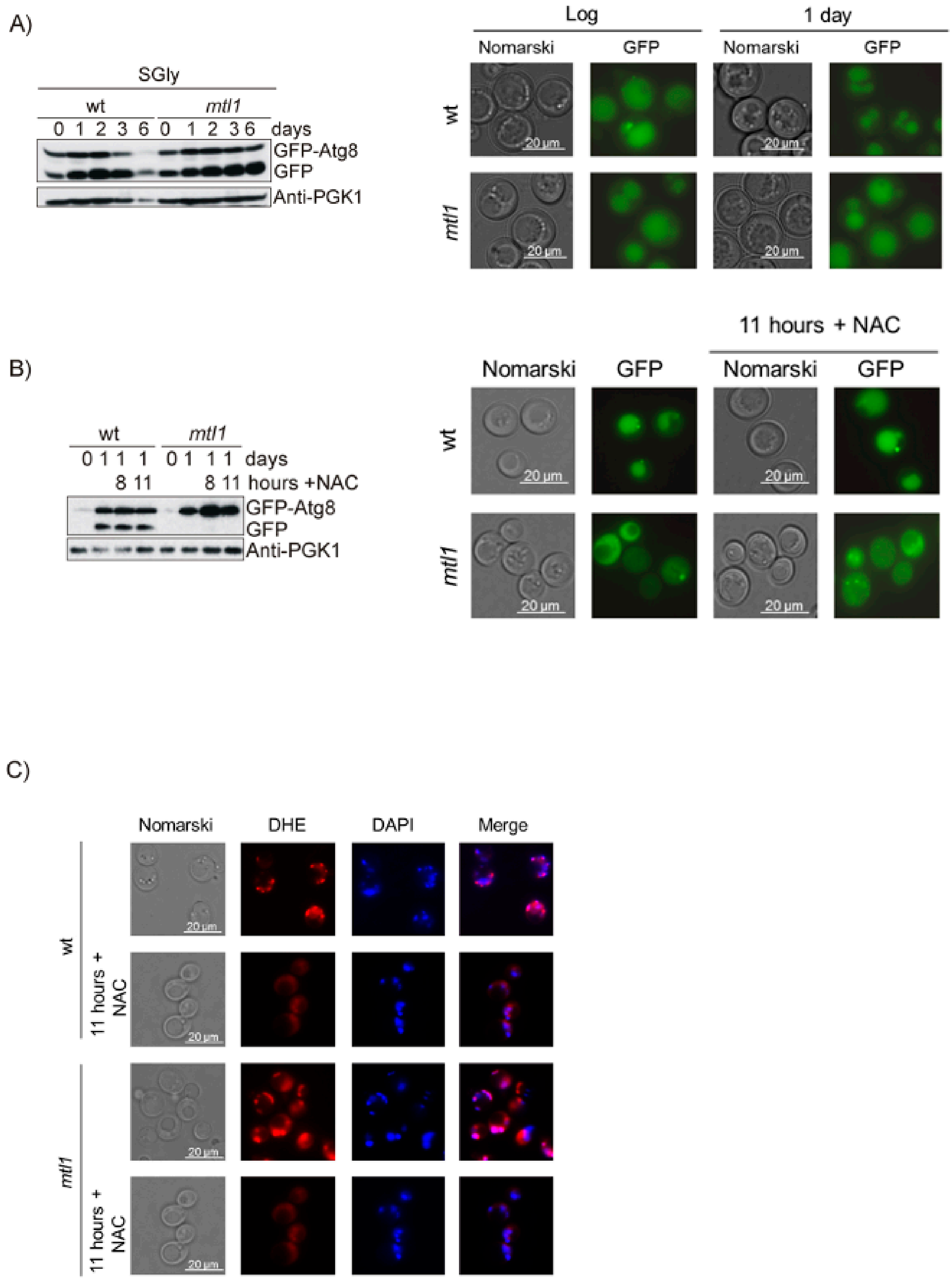

Figure S6. Mtl1 is not deficient in bulk autophagy in respiratory conditions. A) wt and *mtl1* cultures were grown in minimum medium SGly (containing glycerol as unique carbon source) plus amino acids at 30°C to stationary phase for 6 days. Samples were collected at the indicated times to perfor western blot analysis and *in vivo* microscopic

determination as described in (Figure S1A). B) wt and *mtl1* cultures in SD medium growing to 1 day were treated with N-Acetyl cysteine (NAC) 5 mM for 8 and 11 hours. Samples were collected for autophagy determinations as in A. C) wt and *mtl1* cultures were grown in SD medium. At one day of growth, 5mM of NAC was added for 11 hours. Samples were collected and stained with dihydroethidium (DHE) for in vivo visualization of cellular oxidation in the fluorescent microscope.

Figure S7

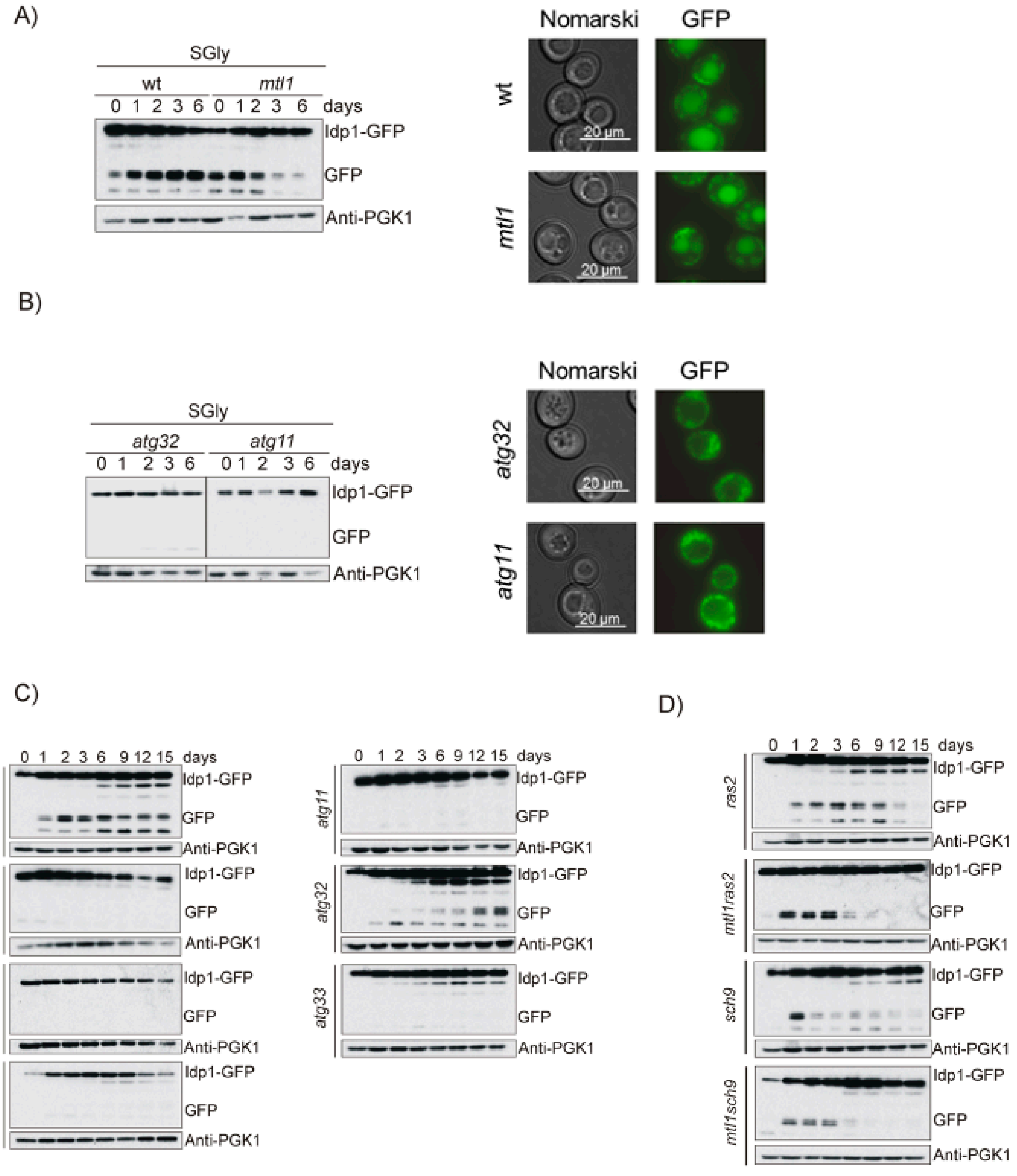

Figure S7. Mtl1 is needed for specific mitochondrial degradation during stationary phase. A) wt and *mtl1* cultures transformed with plasmid Idp1-GFP were grown in SGly media

plus amino acids at 30°C. Samples were taken at the indicated times for *in vivo* observation in the fluorescence microscope and western blot analysis. B) *atg32* and *atg11* cultures transformed with Idp1-GFP were grown in SGly medium plus amino acids at 30°C. Samples were collected at the indicated times for *in vivo* observation in the fluorescence microscope and perform western blot as in A. C) wt, *mtl1, atg1, atg7, atg11, atg32* and *atg33* strains bearing the plasmid Idp1-GFP were grown in SD media at 30ºC for 15 days in continuous shacking. Samples were taken at indicated times to monitor Idp1-GFP cleavage. D) *ras2, mtl1ras2, sch9* and *mtl1sch9* mutants, transformed with Idp1-GFP were treated as in C.
